# Knockout of Tobacco Homologs of *Arabidopsis Multi-Antibiotic Resistance 1* Gene Confers a Limited Resistance to Aminoglycoside Antibiotics

**DOI:** 10.3390/ijms23042006

**Published:** 2022-02-11

**Authors:** Hafizur Rahman, Chika Fukushima, Hidetaka Kaya, Takashi Yaeno, Kappei Kobayashi

**Affiliations:** 1 The United Graduate School of Agricultural Sciences, Ehime University, Tarumi, Matsuyama 790-8566, Japan; hafizbau@gmail.com (H.R.); kaya.hidetaka.hu@ehime-u.ac.jp (H.K.); yaeno@agr.ehime-u.ac.jp (T.Y.); 2 Faculty of Agriculture, Ehime University, Matsuyama 790-8566, Japan; e611044h@mails.cc.ehime-u.ac.jp; 3 Research Unit for Citromics, Ehime University, Matsuyama 790-8566, Japan

**Keywords:** aminoglycoside, CRISPR/Cas9, multi-antibiotic resistance, recessive positive selection, tobacco

## Abstract

To explore a possible recessive selective marker for future DNA-free genome editing by direct delivery of a CRISPR/Cas9-single guide RNA (sgRNA) ribonucleoprotein complex, we knocked out homologs of the *Arabidopsis* *Multi-Antibiotic Resistance 1* (*MAR1*)*/RTS3* gene, mutations of which confer aminoglycoside resistance, in tobacco plants by an efficient *Agrobacterium-*mediated gene transfer. A *Cas9* gene was introduced into *Nicotiana tabacum* and *Nicotiana sylvestris* together with an sgRNA gene for one of three different target sequences designed to perfectly match sequences in both S- and T-genome copies of *N. tabacum* *MAR1* homologs (*NtMAR1h*s). All three sgRNAs directed the introduction of InDels into *NtMAR1h*s, as demonstrated by CAPS and amplicon sequencing analyses, albeit with varying efficiency. Leaves of regenerated transformant shoots were evaluated for aminoglycoside resistance on shoot-induction media containing different aminoglycoside antibiotics. All transformants tested were as sensitive to those antibiotics as non-transformed control plants, regardless of the mutation rates in *NtMAR1h*s. The *NtMAR1h*s–knockout seedlings of the T_1_ generation showed limited aminoglycoside resistance but failed to form shoots when cultured on shoot-induction media containing kanamycin. The results suggest that, like *Arabidopsis MAR1*, *NtMAR1h*s have a role in plants’ sensitivity to aminoglycoside antibiotics, and that tobacco has some additional functional homologs.

## 1. Introduction

In addition to conventional crossbreeding, gene manipulation technologies have been applied to improve crop plants. Different transgenic crops have been bred since the 1980s, but only a few are successful in commercial production. Many countries have legal regulations to constrain the use of genetically modified (GM) crops. Some people are concerned or uncertain about using GM crops as foods [1,2], which could be a limiting factor for the popularization of GM crops. Transgenic technology introduces foreign genes into the crop genome to confer desired traits. By contrast, genome editing technology can modify existing crop genes, as can conventional mutagenesis, but with more precision [3,4]. Therefore, genome editing has the potential to become a significant crop breeding technology, with higher levels of public acceptance than transgenic technology.

Genome editing has widely been applied to several plant species [5]. The clustered regularly interspaced short palindromic repeats (CRISPR)/CRISPR-associated protein 9 (CRISPR/Cas9) has most commonly been used for editing plant genomes among programable endonucleases because it is easy to design single guide RNA (sgRNA) that determines the target sequence [6]. Initially, Cas9 and sgRNA were delivered into plant cells as transgenes through *Agrobacterium*-mediated transformation [7,8,9]. Therefore, legal regulation of GM organisms applies to the resulting plants in some countries. A study demonstrated that plant genomes of *Arabidopsis thaliana*, tobacco, lettuce, and rice could be edited by directly introducing the ribonucleoprotein complex of Cas9 and sgRNA (hereafter RNP) into the protoplasts, followed by plant regeneration from those cells [10]. This method, DNA-free genome editing, is helpful to develop marketable genome-edited crops in those countries with strict GM regulations. Some plants can be marketed after removing any transgene through the genetic cross (so-called null segregants). However, such an approach cannot be used for other crops with high heterozygosity. DNA-free genome editing has now been established in different plant species, but the editing efficiency of DNA-free genome editing is not always high enough.

One possible way to improve the efficiency of DNA-free genome editing is to enrich the cells that received RNP. In genetic transformation, herbicide or antibiotic resistance genes suppress the survival, growth, and redifferentiation of non-transformed cells, thus enriching transformed cells. However, such selective marker genes cannot apply to DNA-free genome editing. The selective marker genes for DNA-free genome editing need to confer resistance to herbicide or antibiotics after being edited by RNPs co-introduced with those for a target gene. One of the candidates for such a selective marker gene encodes acetolactate synthase (ALS), the target enzyme of several different herbicides [11]. One or more amino acid substitutions in the *ALS* gene reportedly confer resistance to herbicides in various plants. Although the *ALS* gene of the herbicide-resistant mutants is dominant over the wild type, a base editor or homologous recombination is necessary to obtain such mutants [12,13,14], alongside much less frequent natural mutation. By contrast, neither a base editor nor homologous recombination is required in another candidate, *MAR1* or *RTS3*, a susceptible gene of multiple aminoglycoside antibiotics [15,16]. Single amino acid substitutions, T-DNA insertions, and hairpin RNA-mediated silencing of *MAR1*/*RTS3* have been shown to confer resistance to kanamycin, tobramycin, gentamycin, streptomycin, amikacin, and apramycin, but not to spectinomycin, paromomycin, hygromycin, and G418. Antibiotic resistant *MAR1*/*RTS3* mutants, although recessive, can be obtained with InDel mutations induced by the CRISPR/Cas9-mediated double-strand DNA cleavage. Thus, the *MAR1*/*RTS3* gene is a good option for a recessive positive selective marker, which could improve the efficiency of DNA-free genome editing, as illustrated in Supplementary Figure S1.

This study tested whether the knockout of *MAR1*/*RTS3* homologs could render tobacco plants resistant to some aminoglycoside antibiotics. We first identified the closest *Nicotiana tabacum* homologs of *MAR1*/*RTS3* in a public database, designed sgRNA, constructed three binary vectors to express Cas9 and different sgRNAs, and transformed *N. tabacum* and *Nicotiana sylvestris* with the vectors. Two out of three sgRNAs induced high mutation rates in the transformants, as demonstrated by the amplicon sequencing analyses. However, none formed shoots on a shoot-induction medium containing kanamycin, gentamycin, or amikacin at a concentration that inhibited the non-transformants’ shoot formation. Although seedlings of T_1_ progenies from selected lines were proven to be knocked out in the *MAR1*/*RTS3* homologs, they exhibited resistance to a low concentration of kanamycin and sensitivity to amikacin, gentamycin, and higher concentrations of kanamycin. The results suggest that tobacco *MAR1/RTS3* homologs have roles in sensitivity to aminoglycoside antibiotics, but their knockout did not confer reliable resistance, enabling selection of the mutated plants in the culture. The results are inconsistent with a recent report that successfully selected tomato shoots by knocking out *MAR1/RTS3* homologs [17]. The role of *MAR1/RTS3* homologs in aminoglycoside susceptibility and their use as a recessive positive selection marker are discussed.

## 2. Results

### 2.1. Identification of Tobacco Homologs of Arabidopsis MAR1/RTS3

**Figure 1:**
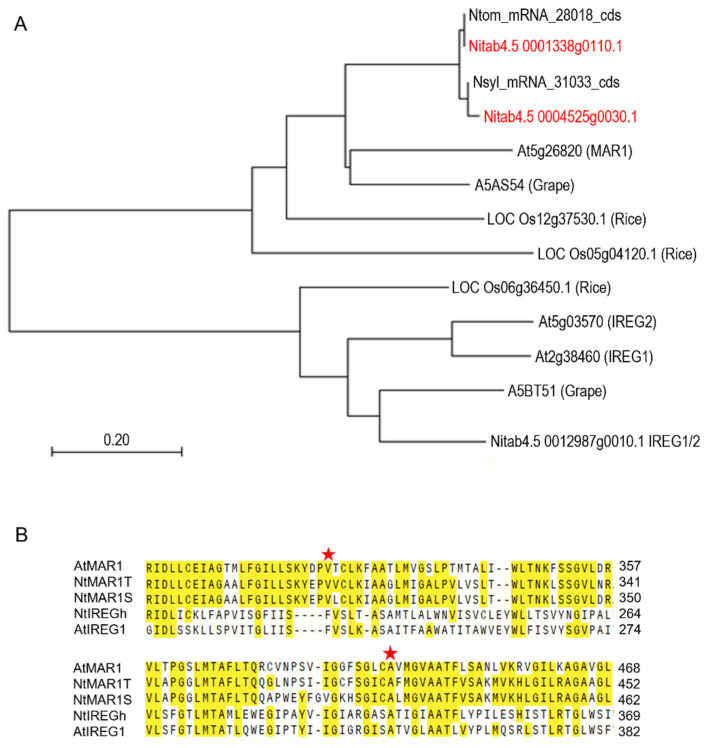
Identification of the *MAR1*/*RTS3* homologs in tobacco. (**A**) A neighbor-joining phylogenetic tree of amino acid sequences of *MAR1*/*RTS3* homologs from *N. tomentosiformis* (Ntom_mRNA_28018_cds), *N. sylvestris* (Nsyl_mRNA_31033_cds), *N. tabacum* (Nitab4.5 0001338g0110.1 and Nitab4.5 0004525g0030.1), *A. thaliana* (At5g26820), *Vitis vinifera* (A5AS54), *Oryza sativa* (LOC Os12g37530.1 and LOC Os05g04120.1), and *IREG1/2* homologs from *Oryza sativa* (LOC Os06g36450.1), *A. thaliana* (At5g03570 (*IREG2*), and At2g38460 (*IREG1*)), *V. vinifera* (A5BT51), and *N. tabacum* (Nitab4.5_0012987g0010.1_*IREG1/2*). (**B**) Alignment of partial amino acid sequences of *A. thaliana MAR1/RTS3* (*AtMAR1*), its *N. tabacum* homologs (*NtMAR1T* and *NtMAR1S*)*, IREG1* of *A. thaliana* (*AtIREG1*) and *N. tabacum* (*NtIREGh*). Conserved amino acids are highlighted by yellow. Five-pointed asterisks denote amino acid residues that are altered in *mar1* and *rts3* mutants.

The nucleotide sequences for tobacco homologs of *Arabidopsis MAR1*/*RTS3* and *IREG1* and *IREG2* identified in tobacco genome databases were evaluated by inferring a phylogenetic tree of deduced amino acid sequences (Figure 1A). The result indicates that Nitab4.5 0001338g0110.1 and Nitab4.5 0004525g0030.1 are the closest homologs of *MAR1* located on the T- and S-genomes, respectively. Both valine (#323 in MAR1) and alanine (#441 in MAR1), which were substituted with methionine and valine in the *rts3-1* and *mar1-1* mutants, respectively [16], are conserved in these tobacco homologs (Figure 1B), supporting their orthology to *Arabidopsis MAR1*. Thus, Nitab4.5 0001338g0110.1 and Nitab4.5 0004525g0030.1 are hereafter called *NtMAR1T* and *NtMAR1S*, respectively.

**Figure 2:**
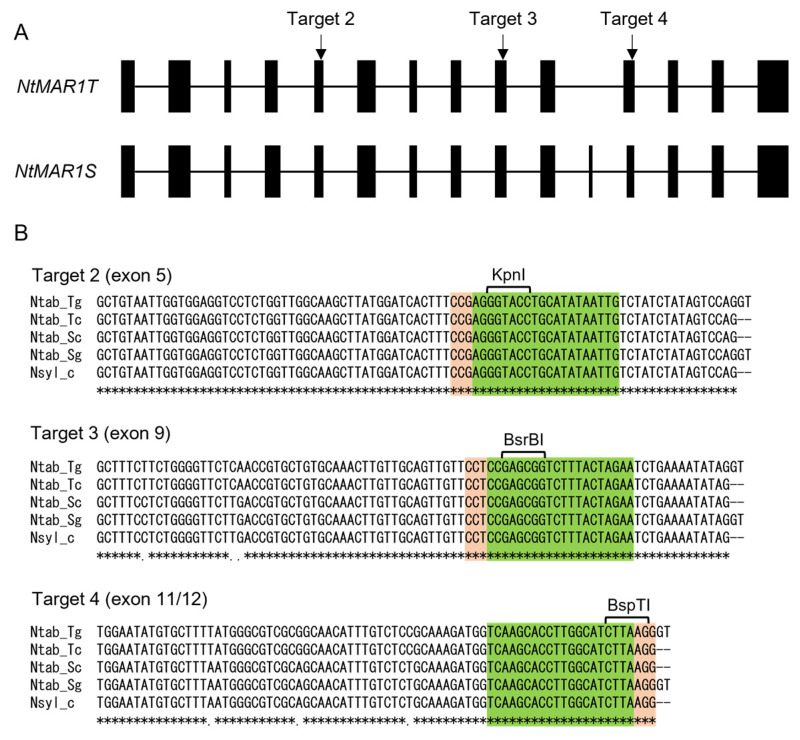
The genome editing targets in tobacco *MAR1*/*RTS3* homologs. (**A**) Scheme of tobacco *MAR1* gene. Exons are shown in filled boxes (14 and 15 exons in *NtMAR1T* and *NtMAR1S*, respectively), and introns with lines (13 and 14 introns in *NtMAR1T* and *NtMAR1S*, respectively). Although the size of exons is shown in proportion to the actual sequence, intron lengths are not. Black arrows denote the location of target sequences. (**B**) Alignment of the genomic and cDNA sequences of *NtMAR1T* and *NtMAR1S* and the cDNA sequence of the *Nicotiana sylvestris* homolog. The PAM and target areas are colored orange and green, respectively. The restriction enzymes used for CAPS analysis and their recognition sites are shown above the alignments. Asterisks denote perfect matches within all five sequences. Dashes indicate gaps in alignments that correspond to introns.

We next aligned the genomic and cDNA sequences of *NtMAR1T* and *NtMAR1S* and the cDNA sequence of the *N. sylvestris* homolog (Supplementary Figure S2) to confirm the sequence identity in the CRISPR/Cas9 targets. *NtMAR1T* and *NtMAR1S* were shown to have 14 and 15 exons (Figure 2A). We designed four target sequences, which perfectly matched within five sequences. We introduced sequences corresponding to three of them, targets 2, 3, and 4 (hereafter called T2, T3, and T3; Figure 2B), respectively, to the binary vector (Supplementary Figure S3).

### 2.2. CRISPR/Cas9-Mediated Mutagenesis of MAR1/RTS3 Homologs in Tobacco Plants

*N. tabacum* and *N. sylvestris* plants were transformed using the binary vectors above, and transformant shoots selected by hygromycin resistance were first tested for mutations in the *MAR1/RTS3* homologs by CAPS analysis, using the restriction enzymes shown in Supplementary Table S2. Based on the CAPS analyses results (Supplementary Figure S4), we selected 10 plant lines each from *N. tabacum* and *N. sylvestris* transformants with high or low mutation rates. They were analyzed by amplicon sequencing to discover the InDel mutation rates and patterns.

**Figure 3:**
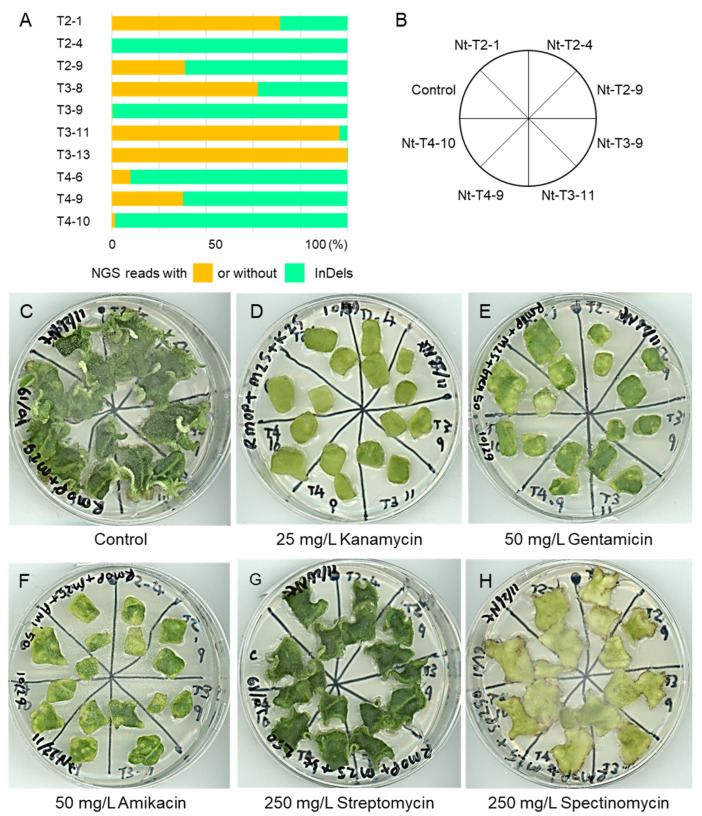
Evaluation of aminoglycoside antibiotic resistance in tissue culture of T_0_ generation of *N. tabacum*. (**A**) indicates the ratio of amplicon sequencing reads with and without InDels at the target sites in selected transgenic lines. Resistance of selected transgenic lines to aminoglycoside antibiotics was evaluated by shoot formation capacity of the leaf pieces from non-transformed plants (Control) and some selected T_0_ transgenic plants placed on the test media (**C**–**H**) as shown in (**B**). The test media were an RMOP medium containing 25 mg/L meropenem alone (C; control plate without aminoglycosides), and those containing 25 mg/L kanamycin (**D**), 50 mg/L gentamycin (**E**), 50 mg/L amikacin (**F**), 250 mg/L streptomycin (**G**), or 250 mg/L spectinomycin (**H**) in combination with 25 mg/L meropenem.

The amplicon sequencing of selected *N. tabacum* lines revealed that the InDel mutation rates ranged from less than 1% to more than 99%, consistent with the CAPS analysis results (Figure 3A and Supplementary Figure S4B–D). Plant lines with higher mutation rates included Nt-T3-11 (96.45%) and Nt-T3-13 (99.95%), those with moderate mutation rates included Nt-T2-1 (71.4%), Nt-T2-9 (31.03%), Nt-T3-8 (61.96%), and Nt-T4-9 (30.19%), and those with low mutation rates were Nt-T2-4 (0.16%), Nt-T3-9 (0.18%), Nt-T4-6 (7.74%), and Nt-T4-10 (1.44%) (Figure 3A, bar graphs and Supplementary Materials). Most of the InDel mutations were likely to knock out the target genes through frameshifts, but some *NtMAR1S* genes in Nt-T3-13 had a single codon deletion, which may not abolish the gene function (Supplementary Figure S5).

**Figure 4:**
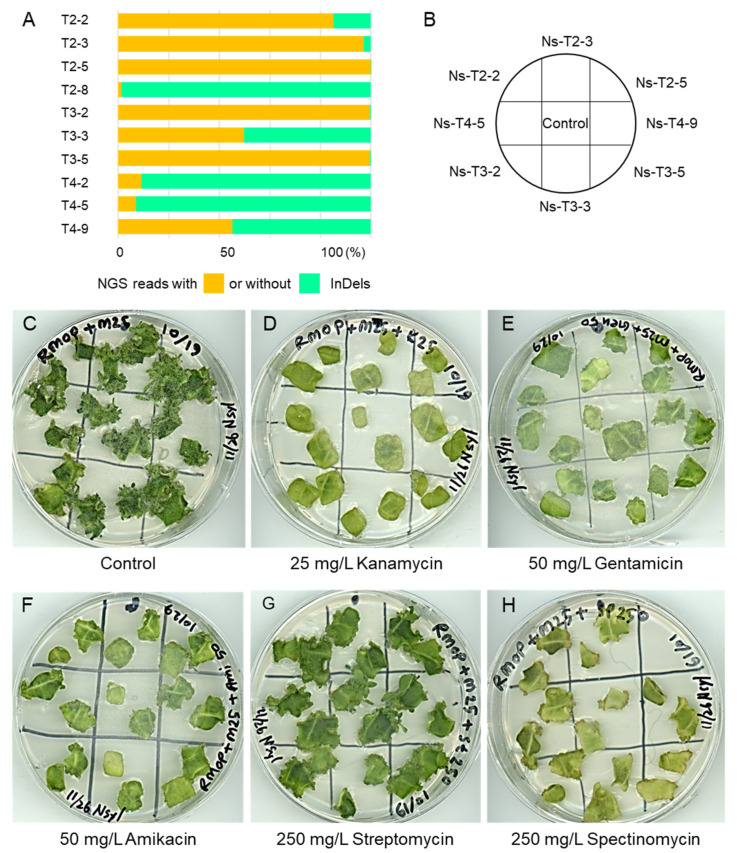
Evaluation of aminoglycoside antibiotic resistance in tissue culture of T_0_ generation of *N. sylvestris*. (**A**), bar graphs showing the ratio of NGS reads with or without InDels at the target sites in the selected transgenic lines. (**B**–**H**), control and some selected transgenic plants were evaluated for their aminoglycoside resistance as described in the caption for Figure 3.

In the transformed *N. sylvestris* plants, InDel mutation rates were higher than in transformed *N. tabacum* lines Figure 3A and Figure 4A and Supplementary Materials). In T2-targeted lines, InDel mutation rates were 85.28%, 97.3%, and 99.97% in Ns-T2-2, -3, and -5, respectively, but as low as 1.29% in Ns-T2-8. Similarly, the mutation rates were 99.64%, 49.88%, and 99.52% in Ns-T3-2, -3, and -5, respectively, in T3-targeted N. sylvestris lines. As in N. tabacum plants, the mutation rates were not very high in T4-targeted N. sylvestris lines, compared to those targeted in T2 or T3, as follows: 9.1%, 7.02%, and 45.01% in Ns-T42, -5, and 9, respectively.

### 2.3. Mutations of Tobacco MAR1/RTS3 Homologs Failed to Confer Aminoglycoside Resistance in Tissue Culture of the T_0_ Generation

Next, we examined aminoglycoside resistance in transgenic plant lines with different mutation rates in the *MAR1/RTS3* homologs. Because we aimed to evaluate whether the knockout of tobacco *MAR1* homologs could serve as a selection marker in future DNA-free genome editing, we tested plants with high and low mutation rates for their shoot regeneration capacity on media containing different aminoglycoside antibiotics. We first determined the minimum effective concentrations of kanamycin, amikacin, gentamycin, streptomycin, and spectinomycin to suppress shoot formation of non-transformed plants. Figure 3B and Figure 4B show the position of leaf pieces on the test media of the control and transgenic plants.

Although all of the *N. tabacum* lines, including the non-transformed control, formed a number of shoots on the control medium containing meropenem but no aminoglycoside (Figure 3C and Figure 4C), none of the *N. tabacum* lines developed shoots in the presence of 25 mg/L kanamycin, 50 mg/L gentamycin, or 50 mg/L amikacin (Figure 3D–F). In the presence of 250 mg/L streptomycin, the leaf pieces stayed green and grew but failed to form shoots (Figure 3G). We included spectinomycin, to which *Arabidopsis mar1* mutants are sensitive, as a kind of control and, as expected, none of the plants formed shoots in its presence (Figure 3H). The results indicate that the knockout of *N. tabacum MAR1* homologs is unlikely to select genome-edited plants in tissue culture. Likewise, *N. sylvestris* did not show evident resistance to any aminoglycosides tested, although we expected shoot formation in the diploid *N. sylvestris* lines with high mutation rates (Figure 4A). They failed to form shoots in the presence of 25 mg/L kanamycin (Figure 4D). In the presence of 50 mg/L gentamycin or 50 mg/L amikacin, a small number of tiny shoots were observed, not only in some transgenic lines but also in the control plants (Figure 4E,F). The responses to two other aminoglycosides of the transgenic plants were also indistinguishable from the control plants (Figure 4G,H). The results indicate that the knockout of the *MAR1* homolog did not confer resistance to aminoglycosides to *N. sylvestris* in tissue culture.

### 2.4. Knockout of Tobacco MAR1/RTS3 Homologs Conferred a Limited Aminoglycoside Resistance in Seedlings

**Figure 5:**
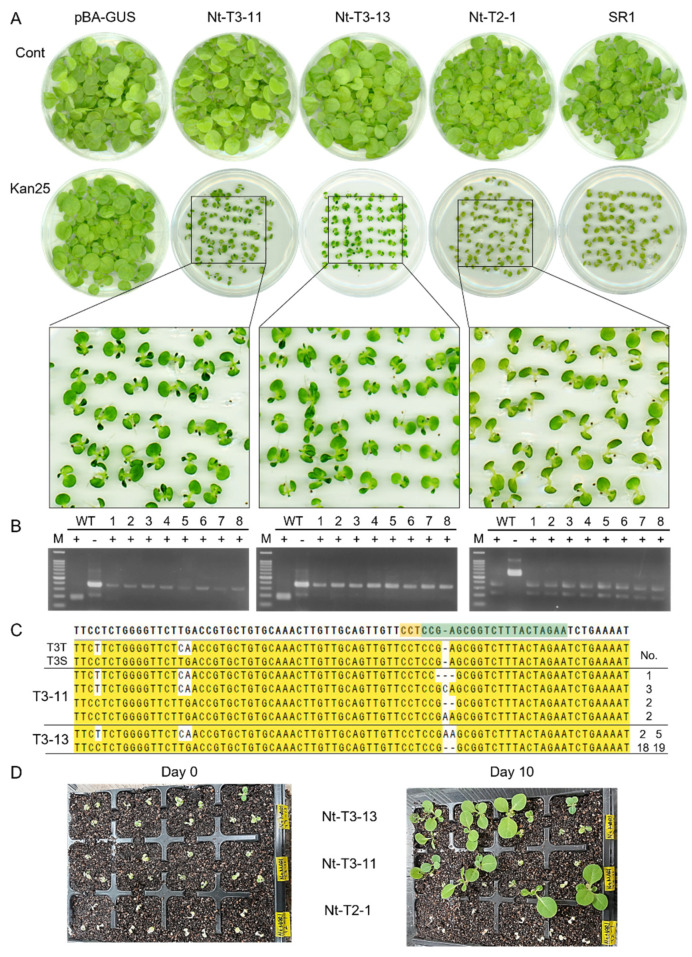
(**A**) limited aminoglycoside resistance of *NtMAR1S*/*NtMAR1T*-knockout *N. tabacum*. (**A**) growth of wild-type (SR1) and T_1_ seedlings of transgenic tobacco lines with a kanamycin resistance gene (pBA-GUS), and candidates of *NtMAR1S*/*NtMAR1T*-knockout plants (T2-1, T3-11, and T3-13) on the media with (Kan25) or without (Cont) 25 mg/L kanamycin. Enlarged images of seedlings from three candidate lines are shown. (**B**) The CAPS analyses of *NtMAR1S*/*NtMAR1T* in three candidate lines. M, Molecular marker; WT, Wild type; 1–8, T_1_ individuals; +, restriction enzyme digested PCR products; -, undigested PCR products. (**C**) Sanger sequencing results of PCR products from a single Nt-T3-11 seedling and two Nt-T3-13 seedlings. The S-genome sequence is shown on the top, with the target sequence in green and the PAM site in yellow highlights. T3T, T-genome sequence encompassing the T3 target sequence; T3S, S-genome sequence encompassing the T3 target sequence; No., the clone numbers for the indicated sequences. (**D**) Growth of seedlings of three candidate lines after 10 days in soil. Seedlings grown for 5 weeks in the presence of 25 mg/L kanamycin were transferred to soil and grown for 10 days. As a control, Nt-T2-1 is used.

Although we failed to detect aminoglycoside resistance in tissue culture of tobacco plants with high mutation rates in *MAR1* homologs, we collected T_1_ seeds from a few *N. tabacum* lines, Nt-T2-1, Nt-T3-11, and Nt-T3-13. We sowed non-transformant T2-1, and T3-11 seeds on an agar media with kanamycin, amikacin, or gentamycin, or without aminoglycoside, to examine the inheritance of mutations in *NtMAR1T* and *NtMAR1S* and confirm the lack of aminoglycoside resistance (Figure 5A and Supplementary Figure S6). In this assay, we included transgenic tobacco that has a kanamycin resistance gene (*nptII*) (Figure 5A and Supplementary Figure S6, pBA-GUS). Kanamycin-resistant positive control plants showed resistance only to kanamycin, and non-transformed negative control plants showed resistance to none of the aminoglycosides tested (Figure 5A and Supplementary Figure S6). The two transgenic lines showed demonstrable resistance only to hygromycin, because they are likely to have a hygromycin resistance gene from the Cas9 expression vector (Supplementary Figure S6). However, we observed tiny true leaves in Nt-T3-11 but not in Nt-T2-1 seedlings, 3 weeks after seed sowing (Figure 5A). The T_1_ seedlings of Nt-T3-13 also showed similarly limited kanamycin resistance (Figure 5A). The CAPS analysis showed that all of the T_1_ seedlings of Nt-T3-11 and Nt-T3-13 with tiny true leaves had mutations in both *NtMAR1T* and *NtMAR1S*, and T_1_ seedlings of Nt-T2-1, which failed to form true leaves, had no mutations and thus served as a control (Figure 5B). Sequencing of PCR products from an individual plant of Nt-T3-11 identified four frame-shifted alleles (Figure 5C), suggesting that Nt-T3-11 seedlings were knocked out in both *NtMAR1T* and *NtMAR1S*. In Nt-T3-13, we detected two frame-shifted alleles in two independent individuals but no wild-type allele, suggesting that Nt-T3-13 seedlings were also knocked out in both *NtMAR1T* and *NtMAR1S* (Figure 5C). We found that more than three-quarters of sequence reads corresponded to S-genome copies and less than a quarter to T-genome copies in Nt-T3-13 (Figure 5C). The cause for these unequal reads remains to be analyzed in detail, but *NtMAR1T* might have been repaired by homologous recombination with *NtMAR1S*. When the seedlings were transferred to the soil after 5 weeks of culture in the presence of 25 mg/L kanamycin and grown for 10 days, some Nt-T3-11 and Nt-T3-13 seedlings grew, but none of the Nt-T2-1 seedlings did (Figure 5D). These results indicate that the knockout of both *NtMAR1T* and *NtMAR1S* confers limited kanamycin resistance to *N. tabacum*, suggesting that they have a role in kanamycin susceptibility of *N. tabacum,* like *Arabidopsis MAR1* does.

### 2.5. Knockout of Tobacco MAR1/RTS3 Homologs Failed to Confer Aminoglycoside Resistance in Tissue Culture

**Figure 6:**
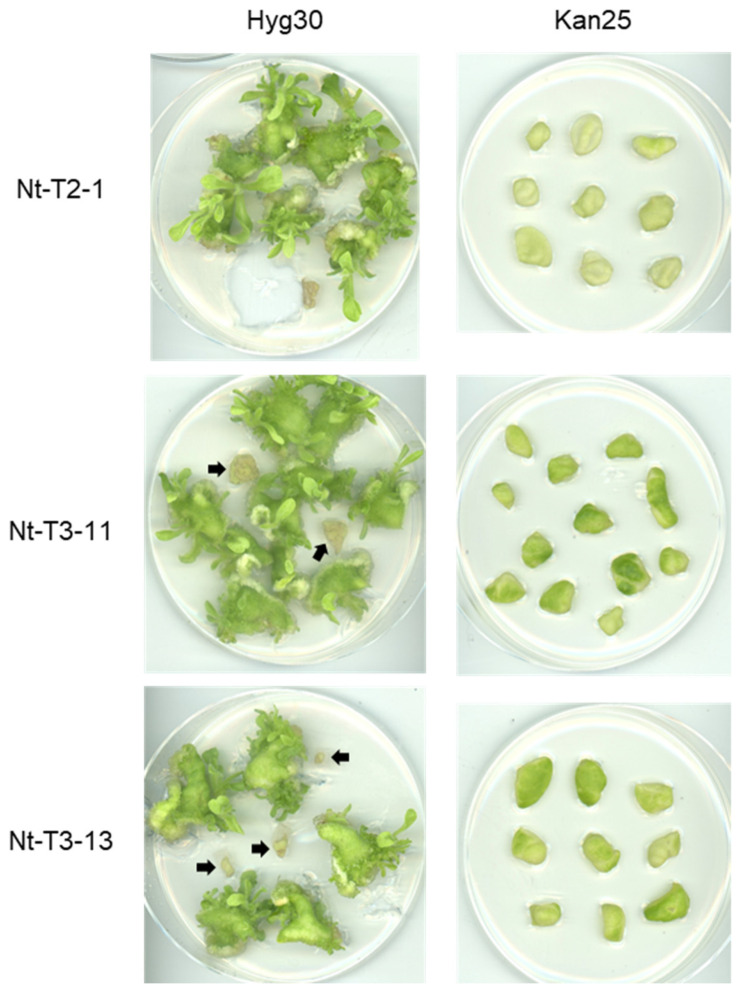
Effect of kanamycin on shoot regeneration of T_1_ seedlings from T2-1, T3-11, and T3-13 lines. Hyg30, shoot induction media containing 30 mg/L hygromycin; Kan25, shoot induction media containing 25 mg/L. Leaf pieces from seedlings grown on antibiotic-free media were tested. Nt-T2-1, which did not have mutated alleles, serves as a control. Arrows indicate hygromycin sensitive leaf pieces (see main text).

Using the knockout T_1_ individuals, we tested their kanamycin resistance in shoot regeneration again to verify the effect of the knockout of the *MAR1*/*RTS3* homologs. As described above, Nt-T2-1 T_1_ individuals did not have any mutations in the target site and therefore, served as a control. Most leaf pieces derived from T_1_ individuals of all three lines, Nt-T2-1, Nt-T3-11, and Nt-T3-13, formed shoots on the RMOP medium containing 30 mg/L hygromycin but some failed, indicating the segregation of the transgene in T_1_ individuals (Figure 6, Hyg30, arrows). On the RMOP medium containing 25 mg/L kanamycin, leaf pieces from T_1_ seedlings of Nt-T2-1 with the wild-type genotype in *MAR1*/*RTS3* homologs failed to form shoots as expected, and leaf pieces from T_1_ seedlings of the knockout lines, Nt-T3-11 and Nt-T3-13, did not form shoots either (Figure 6, Kan25). The results indicate that the knockout of *MAR1*/*RTS3* homologs could not render tobacco plants in tissue culture resistant to kanamycin, despite conferring limited resistance to the seedlings.

## 3. Discussion

Antibiotic selection has been widely used in gene manipulation of diverse organisms, including plants and animals. Unlike animal cell transformation, in which only antibiotics affecting eukaryotic genetic systems such as hygromycin or G418 have been used, plant transformation has successfully used some aminoglycoside antibiotics that target only prokaryotic ribosomes, including kanamycin. Plants’ susceptibility to aminoglycoside antibiotics was one of the catalysts for the discovery of organellar genetic systems. However, the mechanism underlying this susceptibility had not been understood until the analysis of two antibiotic-resistant *Arabidopsis thaliana* mutants, *rts3-1* and *mar1-1* [15,16].

Identification of the causal gene of *rts3-1*, At5g26820 encoding a transporter-like protein, analysis of its T-DNA insertion mutant, and expression of its hairpin RNA allowed scientists to suggest that the encoded protein would transport kanamycin into chloroplasts [15]. The causal gene of *mar1-1*, which was resistant to other aminoglycoside antibiotics, including amikacin and gentamycin, was proven to be the same gene [16]. Overexpression in plants and transgenic expression in yeasts of the *MAR1*/*RTS3* gene increased susceptibility to aminoglycoside antibiotics, and the protein product was localized to the chloroplast envelope [16]. Furthermore, gentamycin uptake assay in isolated chloroplasts has demonstrated that a MAR1/RTS3 transporter-like protein has a role in aminoglycoside uptake by chloroplasts [16]. Although these studies have established the importance of chloroplast uptake in the susceptibility of *Arabidopsis* to aminoglycoside antibiotics, there had been no reports in any other plant species until a recent report of the regeneration of kanamycin-resistant tomato shoots, caused by disrupting *SlyMAR1*, the tomato homolog of *MAR1*/*RTS3* gene [17].

In tobacco, the present study has successfully knocked out the *MAR1/RTS3* homologs, *NtMAR1T* and *NtMAR1S*, and found that the knockout plants show limited but evident kanamycin resistance. Although we failed to observe resistance to other aminoglycoside antibiotics in those knockout plants, the present results demonstrate that *NtMAR1T* and *NtMAR1S* have at least a partial role in the aminoglycoside susceptibility of tobacco plants. Observation of similar aminoglycoside resistance in distantly related *Arabidopsis* and tobacco suggests that *MAR1/RTS3* homologs are responsible for aminoglycoside susceptibility in a wide range of plants. This supports the theory that aminoglycoside susceptibility in plants relies on uptake of the antibiotics by the chloroplast, which Conte et al. experimentally demonstrated [16]. A recent study has shown that the MAR1/FPN3 protein is dual-targeted to plastids and mitochondria [18]. It is likely that *Arabidopsis MAR1/RTS3* has a role in aminoglycoside uptake into both organelles with prokaryotic genetic systems.

Unlike the *Arabidopsis* mutants, *rts3-1* and *mar1-1*, *NtMAR1T*/*NtMAR1S* knockout plants showed little resistance to kanamycin at a low concentration, and growth of the knockout plants was severely retarded when compared to plants with *nptII*. The low-level kanamycin resistance likely resulted from a limited uptake of kanamycin into the chloroplasts and mitochondria of those plants, suggesting that tobacco plants have alternative pathways of kanamycin uptake. Although these alternative pathways remain to be studied, the *NtMAR1T*/*NtMAR1S* knockout plants generated in this study would facilitate searching for the genes or proteins involved in kanamycin uptake of plastids and mitochondria.

Conte et al. demonstrated that the overexpression of the *MAR1* gene resulted in the development of chlorosis, which could be reversed by adding Fe-EDTA to a three-times higher concentration than the regular MS medium [16]. Based on this finding, it was proposed that the MAR1 protein has a role in chloroplast iron homeostasis. Furthermore, extrapolating the possible use of inward polyamine transporter by aminoglycoside antibiotics [19], it was suggested that MAR1 protein transports the polyamine iron chelator, nicotianamine, in nature [20]. Indeed, overexpression of a polyamine-induced protein with a spermidine transport function reportedly increased *Escherichia coli* aminoglycoside sensitivity [21], which is similar to MAR1-overexpressing plants showing hypersusceptibility to kanamycin [16]. A recent study has demonstrated that MAR1/FPN3 has a role in exporting iron ions from plastids and mitochondria [18], which contradicts the nicotianamine import model proposed by Conte and Lloyd [20]. The mode of MAR1 action in aminoglycoside susceptibility remains unclear. Our *NtMAR1T*/*NtMAR1S* knockout tobacco could facilitate biochemical analysis of isolated chloroplasts and further our understanding of the underlying mechanism of MAR1-mediated aminoglycoside susceptibility.

As Aufsatz et al. pointed out, *MAR1*/*RTS3* could provide a novel strategy to confer kanamycin resistance [15]. Indeed, Rinne et al. showed successful regeneration of kanamycin-resistant shoots of tomato plants by disrupting *SlyMAR1* [17]. In contrast, we did not observe shoot regeneration on the RMOP medium from T_0_ tobacco plant leaves with high mutation rates in *NtMAR1T/NtMAR1S* genes or T_1_ plants with no wild-type allele of *NtMAR1T*/*NtMAR1S* genes. Furthermore, *NtMAR1T*/*NtMAR1S*-knockout individual plants showed only limited resistance to low kanamycin concentrations. Although the phenotype of *SlyMAR1* knockout tomato plants remains to be reported, these results collectively suggest that aminoglycoside transport by MAR1 proteins is opportunistic, as has been proposed [20], and their contribution to aminoglycoside susceptibility depends on the plant species. Using *MAR1/RTS3* homologs in plant selection for DNA-free genome editing would only be successful in a limited number of plant species.

## 4. Materials and Methods

### 4.1. Identification of Tobacco Homologs of Arabidopsis MAR1/RTS3

We obtained nucleotide sequences for tobacco homologs of *Arabidopsis MAR1*/*RTS3* by performing a BLAST search of tobacco genome databases (*N. tabacum* v1.0, *N. sylvestris*, and *N. tomentosiformis* in https://solgenomics.net/organism/Nicotiana_tabacum/genome; accessed on 16 March 2020) [22] using the amino acid sequence of *Arabidopsis MAR1*/*RTS3* (AT5G26820) as a query. We similarly obtained the nucleotide sequence of an *N. tabacum* homolog of *Arabidopsis IREG1* and *IREG2*, paralogs of *MAR1*. The nucleotide and amino acid sequences were aligned using GENETYX ver. 8 (GENETYX Co.; Tokyo, Japan) with a manual adjustment of exon–intron junctions. The phylogenetic tree was inferred with amino acid sequences of the homologs from tobacco and other plants, described previously using MEGA7 [23].

### 4.2. Vector Construction

Targets for CRISPR/Cas9-mediated genome editing were selected. Target candidates were searched using the CCTop—CRISPR/Cas9 target online predictor [24], using the *NtMAR1S* (Nitab4.5 0004525g0030.1) cDNA sequence as the target gene and the *Nicotiana tabacum BX* genome for examining off-targets. We selected target sequences that were perfectly conserved within the S- and T-genome sequences (Supplementary Figure S2) from those recommended by CCTop. Expression vectors for the *Cas9* gene from *Streptococcus pyogenes* (*SpCas9*) under the control of parsley UBIQUITIN promoter (Supplementary Figure S3) have been described previously [25,26]. In a high copy number plasmid, annealed double-stranded oligonucleotides corresponding to sgRNA sequences (Supplementary Table S1) were inserted between the AtU6 promoter and sgRNA scaffold sequence. The sgRNA expression units were excised by PacI and AscI and inserted into corresponding sites of the expression vector above.

### 4.3. Plant Transformation

*N. tabacum* cv. Petit Havana SR1 and *N. sylvestris* plants grown aseptically were transformed by *Agrobacterium* (GV3130:pMP90) harboring the plasmids above, as described previously. Transformants were selected on a RMOP medium [27] containing 30 mg/L hygromycin and 25 mg/L meropenem. Shoots were rooted on a vitamin- and phytohormone-free MS medium containing the same antibiotics. Selected plant lines were transferred to a pot containing a commercial soil mix (SuperMix A, Sakata seeds, Yokohama, Japan). Plants were grown or cultured throughout the experiments under 16 h-light/8 h-dark at 25 °C and 45–65% relative humidity.

### 4.4. Mutation Detection by Cleaved Amplified Polymorphic Sequences (CAPS) Analysis and Sequencing

Crude genomic DNA was extracted as described previously. Leaf pieces of about 4–10 mm^2^ were disrupted in 100 μL EK solution (10% (*v*/*v*) Edwards Solution {200 mM Tris-HCl (pH 7.5), 25 mM EDTA (pH 8.0), 250 mM NaCl, 0.5% SDS}, 90% (*v*/*v*) TE Buffer (10 mM Tris-HCl pH 7.5, 1 mM EDTA); [28,29]. DNA fragments encompassing each target were amplified by PCR using KOD-FX neo (TOYOBO, Osaka, Japan) and CAPS primers listed in Supplementary Table S1. PCR products were digested with the indicated restriction enzyme at 37 °C for 36 h for CAPS analysis. PCR products from selected plant lines of the T_0_ generation were reamplified using AmpSeq primers (Supplementary Table S1), and 300-bp paired-end sequences were collected by the Taniguchi Dental Clinic Oral Microbiome Center (Takamatsu, Japan) using MiSeq (Illumina, San Diego, CA, USA). The sequence data were analyzed using CRISPResso 2 [30]. PCR products from T_1_ generation plants were cloned using a Zero Blunt TOPO PCR cloning kit (Thermo Fisher Scientific, Waltham, MA, USA) and sequenced.

### 4.5. Evaluation of Antibiotic Resistance

For evaluating antibiotic resistance in the T_0_ generation, leaves of selected T_0_ plants with high or low mutation rates were cut into approximately 1 cm^2^ pieces and placed on an RMOP medium [27] (25 mL per 90 mm diameter petri dish) containing 25 mg/L meropenem and aminoglycoside antibiotics (25 mg/L kanamycin, 50 mg/L gentamycin, 50 mg/L amikacin, 250 mg/L streptomycin, or 250 mg/L spectinomycin). An RMOP medium containing only meropenem served as a control. The petri dish was sealed with Labopita film (Aglis Co., Ltd., Yame, Japan). Shoot formation was monitored after 3 weeks of culture on the same medium. The medium was sufficient for 3 weeks of culture under our culture room conditions, as described in Section 4.3, as manifested by vigorous shoot formation on the control plates. To evaluate the antibiotic resistance of the T_1_ seedlings, seeds of the T_1_ generation from selected plant lines were sterilized with 10-fold diluted household bleach for 5 min, washed 5 times with autoclaved water, and sown on the vitamin- and phytohormone-free half-strength MS medium (25 mL medium per 90 mm diameter petri dish) containing 25, 50, or 100 mg/L kanamycin, 40 or 100 mg/L gentamycin, or 50 or 100 mg/L amikacin. Antibiotic-free media served as a control. The growth of plants was monitored after 3 weeks of culture and the plants were subjected to molecular analysis. In the repeated experiments, media containing 25 mg/L kanamycin or no antibiotics were used. A control kanamycin-resistant transgenic line was transformed in the same way but using *Agrobacterium* harboring pBA-GUS that has a *nptII* plant selective marker gene derived from pBI121. Some transgenic seedlings were transferred to the commercial soil mix described above after 5 weeks of culture in the presence of 25 mg/L kanamycin and grown under 16h-light/8h-dark at 25 °C for 10 days. To evaluate the antibiotic resistance of the T_1_ seedlings in tissue culture, aseptically grown seedlings were cut into approximately 1 cm^2^ pieces and placed on an RMOP medium containing 25 mg/L kanamycin. An RMOP medium containing 30 mg/L hygromycin served as a control.

## 5. Conclusions

In this study, we successfully knocked out *NtMAR1T*/*NtMAR1S*, tobacco homologs of the *MAR1/RTS3* gene of *Arabidopsis* that is the causal gene of recessive aminoglycoside-resistant mutants [15,16]. However, the knockout tobacco seedlings showed limited aminoglycoside resistance, and their leaves showed no aminoglycoside-resistant shoot regeneration in tissue culture. The results indicate the role of *NtMAR1T*/*NtMAR1S* in aminoglycoside susceptibility. However, the knockout of *NtMAR1T*/*NtMAR1S* is unlikely to be suitable as a selective marker in the future DNA-free genome editing of tobacco, unlike in *Arabidopsis* and tomato [17].

## Data Availability

The data supporting the findings of this study are available within the article and Supplementary Materials.

## Supplementary Materials

The following are available online at https://www.mdpi.com/article/10.3390/ijms23042006/s1.
