# Supplementary material for: Knockout of Tobacco Homologs of Arabidopsis Multi-Antibiotic Resistance 1 Gene Confers a Limited Resistance to Aminoglycoside Antibiotics
Source: Int J Mol Sci. 2022 Feb 11;23(4):2006. doi: 10.3390/ijms23042006 (PMC8878083; doi:10.3390/ijms23042006)
Supplement: Supplementary file 1 [file ijms-23-02006-s001.zip › ijms-1545506-supplementary.pdf]

Supplementary materials for

Knockout of Tobacco Homologs of *Arabidopsis Multi-Antibiotic Resistance 1* Gene Confers a Limited Resistance to Aminoglycoside Antibiotics

Hafizur Rahman, Chika Fukushima, Takashi Yaeno, Hidetaka Kaya, and Kappei Kobayashi

Supplementary Table S1. Oligodeoxynucleotides used in this study

Primer names	Sequences (5'-> 3')	Usage [a]
sgNtMAR1-T2-up	ATTGCAATTATATGCAGGTACCCT	Target [b]
sgNtMAR1-T2-low	AAACAGGGTACCTGCATATAATTG	
sgNtMAR1-T3-up	ATTGTTCTAGTAAAGACCGCTCGG	Target
sgNtMAR1-T3-low	AAACCCGAGCGGTCTTTACTAGAA	
sgNtMAR1-T4-up	ATTGTCAAGCACCTTGGCATCTTA	Target
sgNtMAR1-T4-low	AAACTAAGATGCCAAGGTGCTTGA	
T2CAPS-F	TGCATTCGAACAGCTTTATATGCT	CAPS
T2AS-F	AATGGCTTTCGTTGCTAAGG	AmpSeq
T2CAPS&AS-R	AACAGCTCCCGCTAGAACAA	CAPS/AmpSeq
T3CAPS&AS-F	CGGTTTGATGATTGGGGCAC	CAPS/AmpSeq
T3CAPS&AS-R	TGCTTGCTGGAAGAACAGGT	CAPS/AmpSeq
T4CAPS&AS-F	GGGAACATCACTGTAATGATTTCAGG	CAPS/AmpSeq
T4CAPS&AS-R	ACGGGAGTCTGCTGAGAAAG	CAPS/AmpSeq

[a] Usage of the oligonucleotides. Target, to provide target sequence to the genome-editing vector; CAPS, to amplify the genomic regions encompassing the target sites for CAPS analysis; AmpSeq, to amplify the genomic regions encompassing the target sites for amplicon sequencing.

[b] Oligonucleotide with -up was annealed with its counterpart oligonucleotide with -low to introduce target sequence between the AtU6 promoter & gRNA scaffold sequence.

Supplementary Table S2. Restriction enzymes used in CAPS analyses, amplified product sizes before and after digestion, and reaction conditions in this study

Target	Enzyme	PCR product (bp)	Digested products (bp)	Reaction condition
T2	KpnI	T596/S606 [a]	T362/S372+234	37°C, 36.0 hrs.
T3	BsrBI	381	203+178	37°C, 36.0 hrs.
T4	BspTI	323	128+195	37°C, 36.0 hrs.

[a] Length of PCR products from T- and S-genome copies are different and, therefore, separately shown.

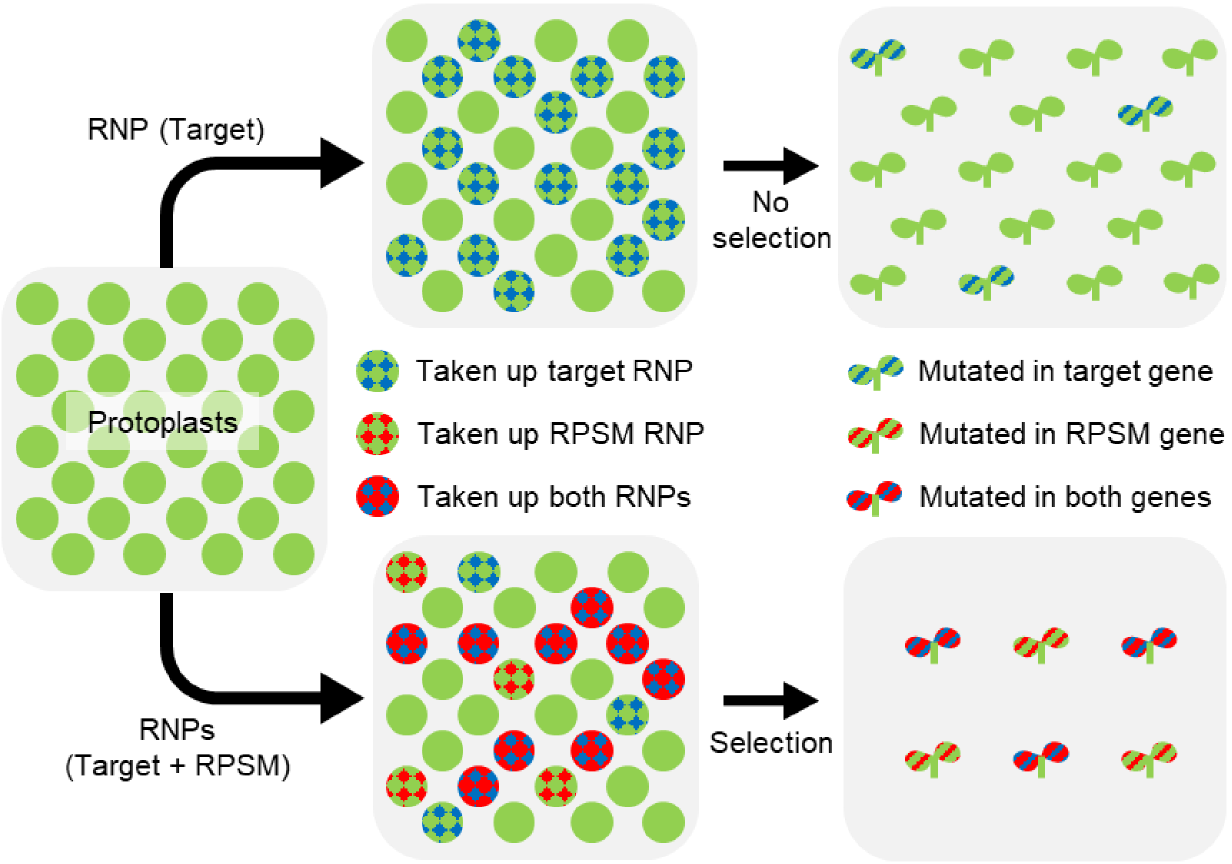

Supplementary Figure S1. A strategy to improve DNA-free genome editing efficiency using a recessive positive selection marker (RPSM). In a non-selective DNA-free genome editing (upper part), RNP for the target gene alone is introduced into plant protoplasts. Some transfected protoplasts take up the RNP (checked blue), get mutated in the target gene, and some of them are regenerated into plants (blue striped). When protoplasts were introduced with RNPs to both target and RPSM genes (lower part), some protoplasts take up both RNPs and get mutated in both genes (checked red and blue). Regenerated plants with RPSM gene mutation grow on a selective medium, enriching the plants with the target gene mutation (red and blue striped).

```
Nitab4.5_0001338_Tg     1:ATGGCGGTTGTTGTTTCTCACGCTTTCCTTGCTGTTCGTCAAGCTCCGTTTACGTCGTCGTCTTCCTTTAGAATACGCCCTTGCTTCTCTTCTCGTCGAT 100
Nitab4.5_0001338_Tc     1:ATGGCGGTTGTTGTTTCTCACGCTTTCCTTGCTGTTCGTCAAGCTCCGTTTACGTCGTCGTCTTCCTTTAGAATACGCCCTTGCTTCTCTTCTCGTCGAT 100
Nitab4.5_0004525_Sc     1:ATGGCGGTTGTTGTTTCTCACGCTTTCCTTGCTGTTCGTCAAGTTCCGTTCATGTCGTCGTCTTCCTTTAGAATACGCCCTTGCTTCTCTTCTCGTCGAT 100
Nitab4.5_0004525_Sg     1:ATGGCGGTTGTTGTTTCTCACGCTTTCCTTGCTGTTCGTCAAGTTCCGTTCATGTCGTCGTCTTCCTTTAGAATACGCCCTTGCTTCTCTTCTCGTCGAT 100
NsylMAR1h-c31033        1:ATGGCGGTTGTTGTTTCTCACGCTTTCCTTGCTGTTCGTCAAGTTCCGTTCACGTCGTCGTCTTCCTTTAGAATACGCCCTTGCTTCTCTTCTCGTCGAT 100
                          *******************************************.******.*.***********************************************

Nitab4.5_0001338_Tg   101:GGTTAACTCCTCCTCGCTTTACCCCTCAAAGGTGCCACCTTTATGCTTTTATTTTAAGCTCTTGACATTCTGAATCACTGATTTGGTACTGTATATCTGC 200
Nitab4.5_0001338_Tc   101:GGTTAACTCCTCCTCGCTTTACCCCTCAAAG--------------------------------------------------------------------- 131
Nitab4.5_0004525_Sc   101:GGTTAACTACTCCTCGCTTTACCCCTCAAAG--------------------------------------------------------------------- 131
Nitab4.5_0004525_Sg   101:GGTTAACTACTCCTCGCTTTACCCCTCAAAGGTGCCTCCTTTATGCTTTTATTTTAAGCTCTTGACATTCTGAATCACTGATTTGGTACTGTATATCTGC 200
NsylMAR1h-c31033      101:GGTTAACTACTCCTCGCTTTACCCCTCAAAG--------------------------------------------------------------------- 131
                          ********.**********************

Nitab4.5_0001338_Tg   201:GTGTGGACATTGTGACTTTACCCCTTTTCCCCAATAAATAGCTTAAATTTGGTCCAATTTTGGTATTCGGACAAAGAATGTAGCACTCGTATAGGATAGT 300
Nitab4.5_0001338_Tc   131:---------------------------------------------------------------------------------------------------- 131
Nitab4.5_0004525_Sc   131:---------------------------------------------------------------------------------------------------- 131
Nitab4.5_0004525_Sg   201:GTGTGGGCATTGTGACTTTACCCCTTTTCCCCAATAAATAGCTTAAATTTGGT-----------ATTCGGACAA-GAATGTAGCACTCGTATAGGATAGT 288
NsylMAR1h-c31033      131:---------------------------------------------------------------------------------------------------- 131


Nitab4.5_0001338_Tg   301:GTGGTCATTGTTGATCATCAGAGATCAAGTGCCTGAGGAAAGTCAATCTACTTTACTACTAATCAAACAGAGGAAAAATAATGTGATATAATGCAAGAGT 400
Nitab4.5_0001338_Tc   131:---------------------------------------------------------------------------------------------------- 131
Nitab4.5_0004525_Sc   131:---------------------------------------------------------------------------------------------------- 131
Nitab4.5_0004525_Sg   289:GTGGTCATTGTTGATCCTCAAGGATCAAATGCCTGAGGAAAGTCAATCTAATTTACTACTAGCCAAACAGAGGAAAAATAATGTGATACAGTGCAAGAGT 388
NsylMAR1h-c31033      131:---------------------------------------------------------------------------------------------------- 131


Nitab4.5_0001338_Tg   401:TGTTGAATTCTTTTCTTTTAATGCAGGGTTGGAAGTTTTAGTTCTAGATGTTCCATAAATACTGACGTCTTAACTGACATTGCAACTGATGAAGAGGTTC 500
Nitab4.5_0001338_Tc   132:--------------------------GGTTGGAAGTTTTAGTTCTAGATGTTCCATAAATACTGACGTCTTAACTGACATTGCAACTGATGAAGAGGTTC 205
Nitab4.5_0004525_Sc   132:--------------------------GGTTGGAAGTTTTAGCTCTAGATGTTCCATAAATACTGACGTCTTAACTGACATTGCAACTGATGAAGAGGTTC 205
Nitab4.5_0004525_Sg   389:TGCTGAATTCTTTGGTTTTAATGCAGGGTTGGAAGTTTTAGCTCTAGATGTTCCATAAATACTGACGTCTTAACTGACATTGCAACTGATGAAGAGGTTC 488
NsylMAR1h-c31033      132:--------------------------GGTTGGAAGTTTTAGCTCTAGATGTTCCATAAATACTGACGTCTTAACTGACATTGCAACTGATGAAGAGGTTC 205
                                                    ***************.**********************************************************

Nitab4.5_0001338_Tg   501:GAGATGATGTTGCAACTGATGATTTCGGCTGTACAGTACCTGTTCTTCATCTCAAATCTGATATCCTGGAAACTGAAGCACTAAATCTGCTAGCTAAGGG 600
Nitab4.5_0001338_Tc   206:GAGATGATGTTGCAACTGATGATTTCGGCTGTACAGTACCTGTTCTTCATCTCAAATCTGATATCCTGGAAACTGAAGCACTAAATCTGCTAGCTAAGGG 305
Nitab4.5_0004525_Sc   206:GGGATGATGTTGCAACTGATGATTTCGGCTGTTCAGTACCTGTTATTCATCTCAAATCTGATATCCTGGAAACTGAAGCACTAAACCTGCTAGCTAAGGG 305
Nitab4.5_0004525_Sg   489:GGGATGATGTTGCAACTGATGATTTCGGCTGTTCAGTACCTGTTATTCATCTCAAATCTGATATCCTGGAAACTGAAGCACTAAACCTGCTAGCTAAGGG 588
NsylMAR1h-c31033      206:GGGATGATGTTGCAACTGATGATTTCGGCTGTTCAGTACCTGTTATTCATCTCAAATCTGATATCCTGGAAACTGAAGCACTAAACCTGCTAGCTAAGGG 305
                          *.******************************.***********.****************************************.**************

Nitab4.5_0001338_Tg   601:CACATTTGTGGACACTCTCCTGACAACATTGCCTGTAGGATTTCGTTATGATTAATTCTACTCAGTCATTTGAGTGCCCCTGTTCCCATCTAACTATCAC 700
Nitab4.5_0001338_Tc   306:CACATTTGTGGACACTCTCCTGACAACATTGCCT------------------------------------------------------------------ 339
Nitab4.5_0004525_Sc   306:CACATTCGTGGACACTCTCTTGACAACATTGCCT------------------------------------------------------------------ 339
Nitab4.5_0004525_Sg   589:CACATTCGTGGACACTCTCTTGACAACATTGCCTGTAGGATTT-GTTATGATTAATTCTACTCAGTCATTTGACTGCCCCTGTTCCCATCTAACTATCAC 687
NsylMAR1h-c31033      306:CACATTCGTGGACACTCTCTTGACAACATTGCCT------------------------------------------------------------------ 339
                          ******.************.**************

Nitab4.5_0001338_Tg   701:TTTGATATTTCTATAATCTATTATTGATGTTGACCAGGTCCTAACAGAGGAGGAGCAGAATATTATTGCTGCAACACCTGCTCATCCAGCAGGGTTATAC 800
Nitab4.5_0001338_Tc   340:-------------------------------------GTCCTAACAGAGGAGGAGCAGAATATTATTGCTGCAACACCTGCTCATCCAGCAGGGTTATAC 402
Nitab4.5_0004525_Sc   340:-------------------------------------GTCCTAACGGAGGAGGAGCAGAATATTATTGCTGCAACACCTGCTCATCCAGCAGGGTTATAC 402
Nitab4.5_0004525_Sg   688:TTTGATTTTTCTATTAT-------TAATGTTGACCAGGTCCTAACGGAGGAGGAGCAGAATATTATTGCTGCAACACCTGCTCATCCAGCAGGGTTATAC 780
NsylMAR1h-c31033      340:-------------------------------------GTCCTAACGGAGGAGGAGCAGAATATTATTGCTGCAACACCTGCTCATCCAGCAGGGTTATAC 402
                                                               ********.******************************************************

Nitab4.5_0001338_Tg   801:GGTTAGTCTTTTCCCT-TAAGTGGAATGACATTTCATATTGTAGTGGTAGAAGCTTAACTTATGGTTAAAAGTGAATGAATTGCTGTCTGCTGTACGTTA 899
Nitab4.5_0001338_Tc   403:G--------------------------------------------------------------------------------------------------- 403
Nitab4.5_0004525_Sc   403:G--------------------------------------------------------------------------------------------------- 403
Nitab4.5_0004525_Sg   781:GGTTAGTCTTTTCCTCTTAGGTGGAGTGGCATTTCATATTAGTGTGGTAGAAGCTTAACTTATGGTTAAAAGTGAATGAATTGCTGTCTGCTGTACGTTA 880
NsylMAR1h-c31033      403:G--------------------------------------------------------------------------------------------------- 403
                          *
```

Supplementary Figure S2 page 1

```
Nitab4.5_0001338_Tg  900:AAATGATGGTAAAGGCATAACCTCAGCCTGGTTTGGAATTAACCAAAGGATACTTCTGTGAAATACTAATACATTGAGTATCTGGTGCAGTCCTCTAGAA 999
Nitab4.5_0001338_Tc  403:---------------------------------------------------------------------------------------------------- 403
Nitab4.5_0004525_Sc  403:---------------------------------------------------------------------------------------------------- 403
Nitab4.5_0004525_Sg  881:AAATGCTGATAAATGCATAACCTCAACCTGGTTTGGAATTAACCAAAGGATACTTCTGTGAAATACCAATACATCGAGAATCTGGTGCGGTCCTCTAGAA 980
NsylMAR1h-c31033     403:---------------------------------------------------------------------------------------------------- 403

Nitab4.5_0001338_Tg 1000:GTTCGTATGATATGAGGTTGAGACATAAATATCTA----------------------------------------------------------------- 1034
Nitab4.5_0001338_Tc  403:---------------------------------------------------------------------------------------------------- 403
Nitab4.5_0004525_Sc  403:---------------------------------------------------------------------------------------------------- 403
Nitab4.5_0004525_Sg  981:GTTCGTATGATATGAGGCTGAGACACAAATATCTAAAAAAAGGGTAGCCCGGTGCACTAAGCTCCCGCTATGGTCCAGGGAAGGGCCAGACCGCAAGGATC 1080
NsylMAR1h-c31033     403:---------------------------------------------------------------------------------------------------- 403

Nitab4.5_0001338_Tg 1034:---------------------------------------------------------------------------------------------------- 1034
Nitab4.5_0001338_Tc  403:---------------------------------------------------------------------------------------------------- 403
Nitab4.5_0004525_Sc  403:---------------------------------------------------------------------------------------------------- 403
Nitab4.5_0004525_Sg 1081:TATTGTACACAGCCTTACCCTTTATTTCTGCAAGAGGCTGTTTCCAAATAGACCCTCGGCATCCTTCCCTTTAAGAACTCCCCACCTTGCTCTTGGGGTT 1180
NsylMAR1h-c31033     403:---------------------------------------------------------------------------------------------------- 403

Nitab4.5_0001338_Tg 1034:---------------------------------------------------------------------------------------------------- 1034
Nitab4.5_0001338_Tc  403:---------------------------------------------------------------------------------------------------- 403
Nitab4.5_0004525_Sc  403:---------------------------------------------------------------------------------------------------- 403
Nitab4.5_0004525_Sg 1181:ATGCTTGCATTAGTTATGCTAAAATATTTCTTATCCATTGTTTGGTTTGATGTCAGACAAAAGGGGTAAGCAACCTCCACTTCCAACCAAGAGGTCGTGA 1280
NsylMAR1h-c31033     403:---------------------------------------------------------------------------------------------------- 403

Nitab4.5_0001338_Tg 1034:---------------------------------------------------------------------------------------------------- 1034
Nitab4.5_0001338_Tc  403:---------------------------------------------------------------------------------------------------- 403
Nitab4.5_0004525_Sc  403:---------------------------------------------------------------------------------------------------- 403
Nitab4.5_0004525_Sg 1281:GTTCGAGTCACCCCAAGAGCAAGGTGGGGAGTTCTTGGAGGGAAGTATGCCGAGGGTCTATTTGGAAACAACCACTCTATCCCAGGGTAGGGGTAAGGTC 1380
NsylMAR1h-c31033     403:---------------------------------------------------------------------------------------------------- 403

Nitab4.5_0001338_Tg 1034:---------------------------------------------------------------------------------------------------- 1034
Nitab4.5_0001338_Tc  403:---------------------------------------------------------------------------------------------------- 403
Nitab4.5_0004525_Sc  403:---------------------------------------------------------------------------------------------------- 403
Nitab4.5_0004525_Sg 1381:TGCGTGCACACTACCCTCCCCAGACCCCACTAGTGGGATTATACTGGGTGGTTATTGTTGTTGTTGTTTGGTTTGATGTATTAAAGCATTGCACAATTTT 1480
NsylMAR1h-c31033     403:---------------------------------------------------------------------------------------------------- 403

Nitab4.5_0001338_Tg 1034:---------------------------------------------------------------------------------------------------- 1034
Nitab4.5_0001338_Tc  403:---------------------------------------------------------------------------------------------------- 403
Nitab4.5_0004525_Sc  403:---------------------------------------------------------------------------------------------------- 403
Nitab4.5_0004525_Sg 1481:TTAAAGAATTGTTTGTTTACAAAAATACCCTCATAAACAGTCCATCACTTTATCTTTTTATAGAAACATATGTTGAGGAATGTTTAATTATGAAAAGATT 1580
NsylMAR1h-c31033     403:---------------------------------------------------------------------------------------------------- 403

Nitab4.5_0001338_Tg 1034:---------------------------------------------------------------------------------------------------- 1034
Nitab4.5_0001338_Tc  403:---------------------------------------------------------------------------------------------------- 403
Nitab4.5_0004525_Sc  403:---------------------------------------------------------------------------------------------------- 403
Nitab4.5_0004525_Sg 1581:TTAAAAAAATTATTTGATTTGTCTACTTATATTGTAGTATAGAACTGAATATTTGTTTATAAAAAAGGAAATATGCTAAGTATTTATTTATCACTTTCTT 1680
NsylMAR1h-c31033     403:---------------------------------------------------------------------------------------------------- 403

Nitab4.5_0001338_Tg 1034:---------------------------------------------------------------------------------------------------- 1034
Nitab4.5_0001338_Tc  403:---------------------------------------------------------------------------------------------------- 403
Nitab4.5_0004525_Sc  403:---------------------------------------------------------------------------------------------------- 403
Nitab4.5_0004525_Sg 1681:GGATTATTAGGCAAAGAAAGTTTAAATTAAAAAATAAAAGGAAAATGAGTTGATTATAAGTGCATTCCAATACGTAGTAATAGAGTACAAATTTATAAAT 1780
NsylMAR1h-c31033     403:---------------------------------------------------------------------------------------------------- 403
```

```
Nitab4.5_0001338_Tg 1035:-----------------------------------------------TTTTCTTTTAATCAGATTTCATATGGTAGATGGGTTATTTGGAGTAACAAAGC 1087
Nitab4.5_0001338_Tc  403:---------------------------------------------------------------------------------------------------- 403
Nitab4.5_0004525_Sc  403:---------------------------------------------------------------------------------------------------- 403
Nitab4.5_0004525_Sg 1781:TTACAATGAAGAAATTGGCAGAGTATAGATATAAATAGCGGGAGCAATTTCAATATCAAACTTTACATCAAACTTGCATTAATATAATAGCATATTTTAA 1880
NsylMAR1h-c31033     403:---------------------------------------------------------------------------------------------------- 403

Nitab4.5_0001338_Tg 1088:AATGTCATTGTACAAGCTGAAGATTTTACTTTTGAGAGCTCCCTAAGAACTCAAATCTTTTATCTTCAGTAACGGTATATATATAGTGTTTCCTACGAAA 1187
Nitab4.5_0001338_Tc  403:---------------------------------------------------------------------------------------------------- 403
Nitab4.5_0004525_Sc  403:---------------------------------------------------------------------------------------------------- 403
Nitab4.5_0004525_Sg 1881:TCAAGCATAAATTTTGAAGGGCAAATTTGTCTTTAACTAATCTAATGCATGCATTGAAACCCATTGCATTGTTAATAACATGGTTTCCTATGCATTAGTT 1980
NsylMAR1h-c31033     403:---------------------------------------------------------------------------------------------------- 403

Nitab4.5_0001338_Tg 1188:GTTATTTTATGTATGCTTTGAGACTTAATCTAACAAGACCACCACTACTGTTGTTGATGATTCATGGAATTCCGTTAGGGTGAAGAACACCCCCCCCCC 1287
Nitab4.5_0001338_Tc  403:---------------------------------------------------------------------------------------------------- 403
Nitab4.5_0004525_Sc  403:---------------------------------------------------------------------------------------------------- 403
Nitab4.5_0004525_Sg 1981:ATGCTTAGGATAATGCTAAATAGGGTTTATAACTGATGCTTGCTTAACTAATGCATATGTTCAAAAAGTGTACCAAACAAGGTATTATTAATATACAAAG 2080
NsylMAR1h-c31033     403:---------------------------------------------------------------------------------------------------- 403

Nitab4.5_0001338_Tg 1288:CCCCACACCCCGCAACCCAAAATTCCCTCTGGCACATCACCAGGGCAATTTGTTTGATAAGTAGTTTGACTTTGAAAACATGACCAGTTAATGATTAGTC 1387
Nitab4.5_0001338_Tc  403:---------------------------------------------------------------------------------------------------- 403
Nitab4.5_0004525_Sc  403:---------------------------------------------------------------------------------------------------- 403
Nitab4.5_0004525_Sg 2081:CTTATGCATGCATTATTCCACAAAACCCCCCGGCACATCACCAGGGTAATTTGTTTGATAAGTAGTTTGACTTTGAAAACATGACCAGTTAATGATTAGT 2180
NsylMAR1h-c31033     403:---------------------------------------------------------------------------------------------------- 403

                                                                         T2CAPS-F
Nitab4.5_0001338_Tg 1388:GAATTATGCCGGTAATCTCTGATCATTTTATAGATTTCTGATGTCAAGTGCATTCGAACAGCTTTATATGCTAGTTGCCTGGCTGGGAATTTGGTGGAAC 1487
Nitab4.5_0001338_Tc  404:-------------------------------------------------------------CTTTATATGCTAGTTGCCTGGCTGGGAATTTGGTGGAAC 442
Nitab4.5_0004525_Sc  404:-------------------------------------------------------------CTTTATATGCTAGTTGCCTGGCTGGGAATTTGGTGGAAC 442
Nitab4.5_0004525_Sg 2181:CGAGTTATGCCGGTAATCTCTGAACATTTTATAGATTTCTGATGTCAATGCATTCGAACAGCTTTATATGCTAGTTGCCTGGCTGGGAATTTGGTGGAAC 2280
NsylMAR1h-c31033     404:-------------------------------------------------------------CTTTATATGCTAGTTGCCTGGCTGGGAATTTGGTGGAAC 442
                                                                                      ***************************************

                                                                                            T2AS-F
Nitab4.5_0001338_Tg 1488:AACTATGGAATTTTGCCTCGCCTGCTGCTATTGCATTGATTCATCCTAGTCTTCTTCCTGTAGCACTAATGGCTTTCGTTGCTAAGGTATTAACTTTTT 1587
Nitab4.5_0001338_Tc  443:AACTATGGAATTTTGCCTCGCCTGCTGCTATTGCATTGATTCATCCTAGTCTTCTTCCTGTAGCACTAATGGCTTTCGTTGCTAAG-------------- 528
Nitab4.5_0004525_Sc  443:AACTTTGGAATATTGCCTCGCCTGCTGCTATTGCATTGATTCATCCTAGTCTTCTTCCTGTAGCACTAATGGCTTTCGTTGCTAAGGTATTAAATTTTAC 542
Nitab4.5_0004525_Sg 2281:AACTTTGGAATATTGCCTCGCCTGCTGCTATTGCATTGATTCATCCTAGTCTTCTTCCTGTAGCACTAATGGCTTTCGTTGCTAAGGTATTAAATTTTAC 2380
NsylMAR1h-c31033     443:AACTTTGGAATATTGCCTCGCCTGCTGCTATTGCATTGATTCATCCTAGTCTTCTTCCTGTAGCACTAATGGCTTTCGTTGCTAAG-------------- 528
                         ****.******.**************************************************************.......  ....

Nitab4.5_0001338_Tg 1588:ACTTTTTTGGATAAGGTAAGATGTTCTATTAATA---GAAATGTATCAA---GGTGCGAAAGAGGTTTGAAAAAGAGCAGGAC-----TTGAATAGAAGT 1676
Nitab4.5_0001338_Tc  528:---------------------------------------------------------------------------------------------------- 528
Nitab4.5_0004525_Sc  543:TTTCTTGGATAAG--------------------------------------------------------------------------------------- 555
Nitab4.5_0004525_Sg 2381:TTTCTTGGATAAGGTAAGATGTTTTATTTTATTAATAGAAAGGTATCAAGAAGGTGCGAAAGAGGTTT-AAAAAGAGCAGGACGGTCCTTGATTAGAAGT 2479
NsylMAR1h-c31033     528:---------------------------------------------------------------------------------------------------- 528

Nitab4.5_0001338_Tg 1677:TACAGGAAGACTAAAGGCTTGAAGATAATTATTTACCTTACATCCTGCTATACTTTGTACAGCTTGCTGTAATTGGTGGAGGTCCTCTGGTTGGCAAGCT 1776
Nitab4.5_0001338_Tc  529:--------------------------------------------------------------CTTGCTGTAATTGGTGGAGGTCCTCTGGTTGGCAAGCT 566
Nitab4.5_0004525_Sc  556:--------------------------------------------------------------CTTGCTGTAATTGGTGGAGGTCCTCTGGTTGGCAAGCT 593
Nitab4.5_0004525_Sg 2480:TACAGGAAGACTAAAGGCTTGAAGATAATTATTTACCTTACATCCTGCTATACTTTGTACAGCTTGCTGTAATTGGTGGAGGTCCTCTGGTTGGCAAGCT 2579
NsylMAR1h-c31033     529:--------------------------------------------------------------CTTGCTGTAATTGGTGGAGGTCCTCTGGTTGGCAAGCT 566
                                                                                       **************************************
```

```
Nitab4.5_0001338_Tg 1777:TATGGATCACTTTCCGAGGGTACCTGCATATAATTGTCTATCTATAGTCCAGGTATCCTTTTCGGGGTGTGTCGCTAAGAAATGGTGTATGCAAAACTTT 1876
Nitab4.5_0001338_Tc  567:TATGGATCACTTTCCGAGGGTACCTGCATATAATTGTCTATCTATAGTCCAG------------------------------------------------ 618
Nitab4.5_0004525_Sc  594:TATGGATCACTTTCCGAGGGTACCTGCATATAATTGTCTATCTATAGTCCAG------------------------------------------------ 645
Nitab4.5_0004525_Sg 2580:TATGGATCACTTTCCGAGGGTACCTGCATATAATTGTCTATCTATAGTCCAGGTATCCTTTTCGGGGTGTGTCGCTAAGGAATGGTGTATGCAAAACTTT 2679
NsylMAR1h-c31033     567:TATGGATCACTTTCCGAGGGTACCTGCATATAATTGTCTATCTATAGTCCAG------------------------------------------------ 618
                         ****************************************************

Nitab4.5_0001338_Tg 1877:TTCCTGAAAGTTTTCTAATACTTCTTTTCTGTCAGACAGCTGCCCAGTTGGTGTCTGTTGGAATGATTATACATGGTCACACTCTTCATCCAACATCTGC 1976
Nitab4.5_0001338_Tc  619:-----------------------------------ACAGCTGCCCAGTTGGTGTCTGTTGGAATGATTATACATGGTCACACTCTTCATCCAACATCTGC 683
Nitab4.5_0004525_Sc  646:-----------------------------------ACAGCTGCCCAGTTGGTGTCTGTTGGAATGATTATACATGGTCATACTCTTCATCCAACATCTGC 710
Nitab4.5_0004525_Sg 2680:TTCCTGCAAGTTTTCTAATACTTCTTTTCTGTCAGACAGCTGCCCAGTTGGTGTCTGTTGGAATGATTATACATGGTCATACTCTTCATCCAACATCTGC 2779
NsylMAR1h-c31033     619:-----------------------------------ACAGCTGCCCAGTTGGTGTCTGTTGGAATGATTATACATGGTCATACTCTTCATCCAACATCTGC 683
                                                            ********************************************.********************

                                                            T2CAPS&AS-R (compl)
Nitab4.5_0001338_Tg 1977:ATCATCTTTACTTTTCCGTCCTTGGTTTGTTGTGCTTGTTCTAGCGGGAGCTGTTGAGAGGCTATCTGGGTTGGCATTGGGGGTCGCAGTGGAGCGTGAT 2076
Nitab4.5_0001338_Tc  684:ATCATCTTTACTTTTCCGTCCTTGGTTTGTTGTGCTTGTTCTAGCGGGAGCTGTTGAGAGGCTATCTGGGTTGGCATTGGGGGTCGCAGTGGAGCGTGAT 783
Nitab4.5_0004525_Sc  711:ATCATCTTTACTTTTCCGTCCTTGGTTTGTTGTGCTTGTTCTAGCGGGAGCTGTTGAGAGGCTATCTGGGTTGGCATTGGGGGTCGCAGTGGAGCGTGAT 810
Nitab4.5_0004525_Sg 2780:ATCATCTTTACTTTTCCGTCCTTGGTTTGTTGTGCTTGTTCTAGCGGGAGCTGTTGAGAGGCTATCTGGGTTGGCATTGGGGGTCGCAGTGGAGCGTGAT 2879
NsylMAR1h-c31033     684:ATCATCTTTACTTTTCCGTCCTTGGTTTGTTGTGCTTGTTCTAGCGGGAGCTGTTGAGAGGCTATCTGGGTTGGCATTGGGGGTCGCAGTGGAGCGTGAT 783
                         ****************************************************************************************************

Nitab4.5_0001338_Tg 2077:TGGGTTGTTTTGGTACTGCTGTTTGAACTTTGTTTTGTCAACTATGTCTCACTTTTTGACTACTTATCGGCTAAGTTGCTCCGACACGGCAGTTTAGGTG 2176
Nitab4.5_0001338_Tc  784:TGGGTTGTTTTG---------------------------------------------------------------------------------------- 795
Nitab4.5_0004525_Sc  811:TGGGTTGTTTTG---------------------------------------------------------------------------------------- 822
Nitab4.5_0004525_Sg 2880:TGGGTTGTTTTGGTACTGCTGTTTGAACTTTGTTTTGTCAACTATATCTTACTTTTTGACTACTTAACGG------------------------------ 2949
NsylMAR1h-c31033     784:TGGGTTGTTTTG---------------------------------------------------------------------------------------- 795
                         ************

Nitab4.5_0001338_Tg 2177:CCGCACCCATGTCGACACGACACTAGTATGGGTGTGGGTATGGGATCCGTACCGGATCTGGTCCAACAATTTTGGGTACTTTAACCACGGCGGACGGAAA 2276
Nitab4.5_0001338_Tc  795:---------------------------------------------------------------------------------------------------- 795
Nitab4.5_0004525_Sc  822:---------------------------------------------------------------------------------------------------- 822
Nitab4.5_0004525_Sg 2949:---------------------------------------------------------------------------------------------------- 2949
NsylMAR1h-c31033     795:---------------------------------------------------------------------------------------------------- 795

Nitab4.5_0001338_Tg 2277:AATTCGAGACGAGATACAATTTGATTCCCGGAATCAGAACCAAAACTAGGGTAGATTTGAAGAAAATAGCATACCTTATCTAGGAAATCAATCCTTTACT 2376
Nitab4.5_0001338_Tc  795:---------------------------------------------------------------------------------------------------- 795
Nitab4.5_0004525_Sc  822:---------------------------------------------------------------------------------------------------- 822
Nitab4.5_0004525_Sg 2949:---------------------------------------------------------------------------------------------------- 2949
NsylMAR1h-c31033     795:---------------------------------------------------------------------------------------------------- 795

Nitab4.5_0001338_Tg 2377:TATCTACAACTGGAGAATAAAAAAAATTCACACTTTACAAGCTATACGTAAGTATTCTATAAAATTTCTCATAATTTAGAGATCTTTTATTTTATTTTAT 2476
Nitab4.5_0001338_Tc  795:---------------------------------------------------------------------------------------------------- 795
Nitab4.5_0004525_Sc  822:---------------------------------------------------------------------------------------------------- 822
Nitab4.5_0004525_Sg 2949:---------------------------------------------------------------------------------------------------- 2949
NsylMAR1h-c31033     795:---------------------------------------------------------------------------------------------------- 795

Nitab4.5_0001338_Tg 2477:TTTATTTTTTTGAATTATTTTTAGCCGGATCCCTGCACCCATATCCGTACTAGGGTCCGTATTTCCGAATCTTAGAATTTACATCTCGAAAGATCCGACC 2576
Nitab4.5_0001338_Tc  795:---------------------------------------------------------------------------------------------------- 795
Nitab4.5_0004525_Sc  822:---------------------------------------------------------------------------------------------------- 822
Nitab4.5_0004525_Sg 2949:---------------------------------------------------------------------------------------------------- 2949
NsylMAR1h-c31033     795:---------------------------------------------------------------------------------------------------- 795

Nitab4.5_0001338_Tg 2577:TCTAGATTTGCACCCGTGTCGGGCACCCGCACCCGTGTCCGAACAACTTCGCACCCGTGTCCGAGCAACTTCGCACCCGTGTCCGAGCAACTTAGCTTAT 2676
Nitab4.5_0001338_Tc  795:---------------------------------------------------------------------------------------------------- 795
Nitab4.5_0004525_Sc  822:---------------------------------------------------------------------------------------------------- 822
Nitab4.5_0004525_Sg 2949:---------------------------------------------------------------------------------------------------- 2949
NsylMAR1h-c31033     795:---------------------------------------------------------------------------------------------------- 795
```

```
Nitab4.5_0001338_Tg 2677:CGGTGCTTGAGCAGTTTCCTAATTTTGAGTCTTGTTTTTACAGTTAGCTGGAACTAATCGCCCAGTTGCTCTTGCTCAAGCAAATGCTATTCTAAGCCGT 2776
Nitab4.5_0001338_Tc  796:-------------------------------------------TTAGCTGGAACTAATCGCCCAGTTGCTCTTGCTCAAGCAAATGCTATTCTAAGCCGT 852
Nitab4.5_0004525_Sc  823:-------------------------------------------TTAGCTGGAACTAATCGCCCAGTTGTTCTTGCTCAAGCAAATGCTATTCTAAGCCGT 879
Nitab4.5_0004525_Sg 2950:---TGCTTGAGCAGTTTCCTAATTTTAAGTCATGTTTTTACAGTTAGCTGGAACTAATCGCCCAGTTGTTCTTGCTCAAGCAAATGCTATTCTAAGCCGT 3046
NsylMAR1h-c31033     796:-------------------------------------------TTAGCTGGAACTAATCGCCCAGTTGCTCTTGCTCAAGCAAATGCTATTCTAAGCCGT 852
                                                                    *************************.******************************

Nitab4.5_0001338_Tg 2777:ATTGACCTCTTATGCGAGGTAATAGGCACTTCAATGACAGTATTTCCCCCTCTTTTGCAGCATAACGATTATGAATGATCTAAACAAATATATTCAGATG 2876
Nitab4.5_0001338_Tc  853:ATTGACCTCTTATGCGAG---------------------------------------------------------------------------------- 870
Nitab4.5_0004525_Sc  880:ATTGACCTCTTATGCGAG---------------------------------------------------------------------------------- 897
Nitab4.5_0004525_Sg 3047:ATTGACCTCTTATGCGAGGTAATAGGCAATTCAATGGCAGCTTTTCCCCCCCTCTTTTGCAGCGTAATGATTATGAATGATCTAAAAAAATATATTCAGA 3146
NsylMAR1h-c31033     853:ATTGACCTCTTATGCGAG---------------------------------------------------------------------------------- 870
                         ******************

Nitab4.5_0001338_Tg 2877:TATTGATATTTTATTTCTATTGAGTTAAGTTTATAAAACAAACTTTACTTGAAATTTGCCGTGATTGTCATTTAAGCTAAATTTGGTCTTTTATGATTTA 2976
Nitab4.5_0001338_Tc  870:---------------------------------------------------------------------------------------------------- 870
Nitab4.5_0004525_Sc  897:---------------------------------------------------------------------------------------------------- 897
Nitab4.5_0004525_Sg 3147:TGTATTGATATTTTGTTTCTATTGAGTTAAGTTTATCAAACATACTTTACTTGAAATTTGCAGTGATTGTCATTTAAGTTAAAAATTTGGTCTTTTATGA 3246
NsylMAR1h-c31033     870:---------------------------------------------------------------------------------------------------- 870

Nitab4.5_0001338_Tg 2977:TCTTATAGCTCTCTC--------TTTTTGGGATAATTGTAGATTGCTGGAGCGGCATTGTTTGGCATTCTTCTGTCTAAATATGAACCAGTTGTATGCTT 3068
Nitab4.5_0001338_Tc  871:-----------------------------------------ATTGCTGGAGCGGCATTGTTTGGCATTCTTCTGTCTAAATATGAACCAGTTGTATGCTT 929
Nitab4.5_0004525_Sc  898:-----------------------------------------ATTGCTGGTGCGGCATTGTTTGGCATTCTTCTGTCTAAATATGAACCAGTTTTATGCTT 956
Nitab4.5_0004525_Sg 3247:TTTATCTTATAGCTCTCTTTTTTTTTCCGGGATAATTTTAGATTGCTGGTGCGGCATTGTTTGGCATTCTTCTGTCTAAATATGAACCAGTTTTATGCTT 3346
NsylMAR1h-c31033     871:-----------------------------------------ATTGCTGGTGCGGCATTGTTTGGCATTCTTCTGTCTAAATATGAACCAGTTTTATGCTT 929
                                                                  ********.******************************************.*******

                                     T3CAPS&AS-F
Nitab4.5_0001338_Tg 3069:AAAAATTGCTGCCGGTTTGATGATTGGGGCACTTCCTGTTTTGGTAAATCTTAGTCCTGGTTCTATTTCATTCTGTGATTTGAAGTTTAGAACACTGTAT 3168
Nitab4.5_0001338_Tc  930:AAAAATTGCTGCCGGTTTGATGATTGGGGCACTTCCTGTTTTG--------------------------------------------------------- 972
Nitab4.5_0004525_Sc  957:AAAAATTGCTGCCGGTTTGATGATTGGGGCACTTCCTGTTTTG--------------------------------------------------------- 999
Nitab4.5_0004525_Sg 3347:AAAAATTGCTGCCGGTTTGATGATTGGGGCACTTCCTGTTTTGGTAAATCTTAGTTCTGGTTCTATTTCATTCTGTGATTTGAAGTTTAGAACACTGTGT 3446
NsylMAR1h-c31033     930:AAAAATTGCTGCCGGTTTGATGATTGGGGCACTTCCTGTTTTG--------------------------------------------------------- 972
                         *******************************************

Nitab4.5_0001338_Tg 3169:AGCACTTAGATGTTATCACCTTTTTTACTAGGTATCTCTGACATGGCTAACCAACAAGCTTTCTTCTGGGGTTCTCAACCGTGCTGTGCAAACTTGTTGC 3268
Nitab4.5_0001338_Tc  973:-------------------------------GTATCTCTGACATGGCTAACCAACAAGCTTTCTTCTGGGGTTCTCAACCGTGCTGTGCAAACTTGTTGC 1041
Nitab4.5_0004525_Sc 1000:-------------------------------GTATCTCTGACATGGCTAACCAACAAGCTTTCCTCTGGGGTTCTTGACCGTGCTGTGCAAACTTGTTGC 1068
Nitab4.5_0004525_Sg 3447:AGCTCTTAAATGTTATCACCTTTTTTACTAGGTATCTCTGACATGGCTAACCAACAAGCTTTCCTCTGGGGTTCTTGACCGTGCTGTGCAAACTTGTTGC 3546
NsylMAR1h-c31033     973:-------------------------------GTATCTCTGACATGGCTAACCAACAAGCTTTCCTCTGGGGTTCTTGACCGTGCTGTGCAAACTTGTTGC 1041
                                                        ********************************.***********..**********************

Nitab4.5_0001338_Tg 3269:AGTTGTTCCTCCGAGCGGTCTTTACTAGAATCTGAAAATATAGGTATCTTCTCTTGAGTTGAGTTACAATGAAGGTGGAAATAGATTTTACTTTTATACT 3368
Nitab4.5_0001338_Tc 1042:AGTTGTTCCTCCGAGCGGTCTTTACTAGAATCTGAAAATATAG--------------------------------------------------------- 1084
Nitab4.5_0004525_Sc 1069:AGTTGTTCCTCCGAGCGGTCTTTACTAGAATCTGAAAATATAG--------------------------------------------------------- 1111
Nitab4.5_0004525_Sg 3547:AGTTGTTCCTCCGAGCGGTCTTTACTAGAATCTGAAAATATAGGTATCTTCTCTTGAGTTGAGTTACAATGAAGGTGGAAATAGATTTTACTTTTATACT 3646
NsylMAR1h-c31033    1042:AGTTGTTCCTCCGAGCGGTCTTTACTAGAATCTGAAAATATAG--------------------------------------------------------- 1084
                         *******************************************

                                                                                                      T3CAPS&AS-R (compl)
Nitab4.5_0001338_Tg 3369:AAGCCTGTTGTTTGTTTTCAGTGAGAGTGGGTCTGGAAGCTATAAAGCATGGATGGTTTGAATATGTCAAACAACCTGTTCTTCCAGCAAGCATAGCCTA 3468
Nitab4.5_0001338_Tc 1085:---------------------TGAGAGTGGGTCTGGAAGCTATAAAGCATGGATGGTTTGAATATGTCAAACAACCTGTTCTTCCAGCAAGCATAGCCTA 1163
Nitab4.5_0004525_Sc 1112:---------------------TGAGAGTGGGTCTGGAAGCTATAAAGCATGGATGGTTTGAATATGTCAAACAACCTGTTCTTCCAGCAAGCATAGCCTA 1190
Nitab4.5_0004525_Sg 3647:GAGCCTGTTGTTTGTTTTCAGTGAGAGTGGGTCTGGAAGCTATAAAGCATGGATGGTTTGAATATGTCAAACAACCTGTTCTTCCAGCAAGCATAGCCTA 3746
NsylMAR1h-c31033    1085:---------------------TGAGAGTGGGTCTGGAAGCTATAAAGCATGGATGGTTTGAATATGTCAAACAACCTGTTCTTCCAGCAAGCATAGCCTA 1163
                                              *******************************************************************************
```

```
Nitab4.5_0001338_Tg 3469:TGTGCTTTTATACTTCAATGTTGTTCTTGCACCTGGTGGTTTAATGACAGCGTTCTTAACACAGCAAGGTAATTTCTGCATTCTAGACTCGTGATATTCT 3568
Nitab4.5_0001338_Tc 1164:TGTGCTTTTATACTTCAATGTTGTTCTTGCACCTGGTGGTTTAATGACAGCGTTCTTAACACAGCAAG-------------------------------- 1231
Nitab4.5_0004525_Sc 1191:TGTGCTTTTGTACTTCAATGTTGTTCTTGCACCTGGTGGTTTAATGACAGCATTCTTAACACAGCAAG-------------------------------- 1258
Nitab4.5_0004525_Sg 3747:TGTGCTTTTGTACTTCAATGTTGTTCTTGCACCTGGTGGTTTAATGACAGCATTCTTAACACAGCAAGGTAATTTCTGCATTCTAGACTCGTGATATTCT 3846
NsylMAR1h-c31033    1164:TGTGCTTTTGTACTTCAATGTTGTTCTTGCACCTGGTGGTTTAATGACAGCATTCTTAACACAGCAAG-------------------------------- 1231
                         *********.*****************************************.****************

Nitab4.5_0001338_Tg 3569:CAACTTTAGATTTTGCTGGGGTCACTAATTGGATTTTGATAATGTAGCTACACTTAATAAACCTACCATTGGCCATAATGATAGCTTTCTTGTTGTCTCC 3668
Nitab4.5_0001338_Tc 1231:---------------------------------------------------------------------------------------------------- 1231
Nitab4.5_0004525_Sc 1258:---------------------------------------------------------------------------------------------------- 1258
Nitab4.5_0004525_Sg 3847:CACCTTTAGATTTTGCTTGTGTCACTAATTGGATTTTGATAATGTAGCTACTCTTAATAAACCTACCAGTGGCTATAATGATAGCTTTCTTGGTGTCTCT 3946
NsylMAR1h-c31033    1231:---------------------------------------------------------------------------------------------------- 1231


Nitab4.5_0001338_Tg 3669:AAGCATGGTTGTTTTGGATATGATGATGGTTTACTTGG---CTCTAAATGAAAACTGATTTGAGATGTATGATCATCTATTTGATTCTTGCAGTGTGCTG 3765
Nitab4.5_0001338_Tc 1231:---------------------------------------------------------------------------------------------------- 1231
Nitab4.5_0004525_Sc 1258:---------------------------------------------------------------------------------------------------- 1258
Nitab4.5_0004525_Sg 3947:AAGCATGGTTGTTTTGGATATGATGATGGTTTACTTGGTGGCTCTACATGAAAACTGATTTGAGATGTATGATCATCTATTTGACTCTTGCAGTGTACTG 4046
NsylMAR1h-c31033    1231:---------------------------------------------------------------------------------------------------- 1231

Nitab4.5_0001338_Tg 3766:AATCTTACCACTTCATGAAACAATTTACAAGCTCATAGAAAGTGTTCAAACATTAGGGTAGACAACCTGGATTGGTTTGTTGGACCAGGACCTATAAGAA 3865
Nitab4.5_0001338_Tc 1231:---------------------------------------------------------------------------------------------------- 1231
Nitab4.5_0004525_Sc 1258:---------------------------------------------------------------------------------------------------- 1258
Nitab4.5_0004525_Sg 4047:AATCTTACCACTTCATGAAACAATTTACAAGCTCATAGAAAGTGCTCAAACATTAGGGTAGACAACCTGGATTGGTTTGTTGGACCATGACCTATAAGAA 4146
NsylMAR1h-c31033    1231:---------------------------------------------------------------------------------------------------- 1231

Nitab4.5_0001338_Tg 3866:ATAAAA--GTTTGAATTGAATCAATGCTTTTTTGCTTCATCAAAAAAAAAGAATCAATGCTTTTGTTTGTTTGAACTGTTGATTATTATTTCATCAATTTG 3963
Nitab4.5_0001338_Tc 1231:---------------------------------------------------------------------------------------------------- 1231
Nitab4.5_0004525_Sc 1258:---------------------------------------------------------------------------------------------------- 1258
Nitab4.5_0004525_Sg 4147:ATAAAAAAGTTTGAATTGAATCAATGCTTTTGTTTGTTTGAACTTTTGATTATTATTTCATCAATTTGAAATTTATATGGTATTTATGAGCTATCCTGGT 4246
NsylMAR1h-c31033    1231:---------------------------------------------------------------------------------------------------- 1231

Nitab4.5_0001338_Tg 3964:AAATTTATATGGTATTTATGAGCTATCCTGGTTTGTCACAGCCCTTGAGGTCTTGAAGAAATTACAGTTAATCCTATTTTTGGAGAGAATACTCATATCT 4063
Nitab4.5_0001338_Tc 1231:---------------------------------------------------------------------------------------------------- 1231
Nitab4.5_0004525_Sc 1258:---------------------------------------------------------------------------------------------------- 1258
Nitab4.5_0004525_Sg 4247:TTGTCACAGCCCTTGAGGTCTTGAAGAAATTACAGTTAATCCTATTTATGGAGAGAAACTCATATCTGTGAAGTGTACTTTTTGTCATGCGGATATATAC 4346
NsylMAR1h-c31033    1231:---------------------------------------------------------------------------------------------------- 1231

Nitab4.5_0001338_Tg 4064:GTGAAGTGTACTTTTTGTCATGCTGATACTAATATACCATATAGAAGGGATGTTCTTAGAAAGAAAAGAAATGTTCCTTCTTTATCTGTAGATTAAGTCG 4163
Nitab4.5_0001338_Tc 1231:---------------------------------------------------------------------------------------------------- 1231
Nitab4.5_0004525_Sc 1258:---------------------------------------------------------------------------------------------------- 1258
Nitab4.5_0004525_Sg 4347:CATATAGAAGGGATGTTCTTAGAAAGAAACGAAATGTTTAACCTTCTTTATCTGTAGATTAAGTCGTTACTGACTAGGAACCGTTAAAAACATTTAGCTG 4446
NsylMAR1h-c31033    1231:---------------------------------------------------------------------------------------------------- 1231

Nitab4.5_0001338_Tg 4164:TTACTGACCAGGAACCTTTGAAAACATTTAGCTGTTGATTGTTATAACATTTTTTTATTTTACTTTTTTGTATGGATGAGGATCTATGATAATTTTGCCC 4263
Nitab4.5_0001338_Tc 1231:---------------------------------------------------------------------------------------------------- 1231
Nitab4.5_0004525_Sc 1258:---------------------------------------------------------------------------------------------------- 1258
Nitab4.5_0004525_Sg 4447:TTGATTGTTATTAACGTTTATTTATTTATTTTTATTTTTTAAATTTTTATGGATGAGGATCTATGATGGTTTTGTCCCAAAATCCTTCTAATGCCTTAAG 4546
NsylMAR1h-c31033    1231:---------------------------------------------------------------------------------------------------- 1231

Nitab4.5_0001338_Tg 4264:CAAAGTCCTTCTAATGCCATAAGATATTCTATTAACAAGTAGCCTAAGCTCTACCCCATAATAACTTGAGATAGTCCCCATCTTTATTTCTAGCTATTCT 4363
Nitab4.5_0001338_Tc 1231:---------------------------------------------------------------------------------------------------- 1231
Nitab4.5_0004525_Sc 1258:---------------------------------------------------------------------------------------------------- 1258
Nitab4.5_0004525_Sg 4547:ATATCCTATTAACAAGTAGCCTAAACTCTACACCATAATAACTTGAGATAGTCCCCATCTTTATTTCTAGATATTTCTCTTGTAGTAATGTATTATCTGG 4646
NsylMAR1h-c31033    1231:---------------------------------------------------------------------------------------------------- 1231
```

```
Nitab4.5_0001338_Tg 4364:GTTGTAGTAATGTATATTTAATATCGGGAAAGAATGTGAGTTGACTCAAAACCTTAAGACAACATCTTTAAATCTTTTTGGATTCCCTTATCTTATCGAA 4463
Nitab4.5_0001338_Tc 1231:---------------------------------------------------------------------------------------------------- 1231
Nitab4.5_0004525_Sc 1258:---------------------------------------------------------------------------------------------------- 1258
Nitab4.5_0004525_Sg 4647:GGAAAGAATGTGAGTTGACTCAAAACCTTAAGACAACATCTTTAAATCTCTTTGGATTCCCTTATCTTATCGAATAGGGGATATTACACGAGCATCTTGA 4746
NsylMAR1h-c31033    1231:---------------------------------------------------------------------------------------------------- 1231

Nitab4.5_0001338_Tg 4464:TATGGGATATTACACGTGCATCTTGCTTATGGGGTTTATATGTGGACTTTAATGCTATTAACGCCCTTATGCATGTTTAACTGATTTTGGGTTGACATGT 4563
Nitab4.5_0001338_Tc 1231:---------------------------------------------------------------------------------------------------- 1231
Nitab4.5_0004525_Sc 1258:---------------------------------------------------------------------------------------------------- 1258
Nitab4.5_0004525_Sg 4747:TTATGGGGTTTATATGTGGACTTTAATGCTATTAACACCCTTATGCATGTTTAACTGATTTTGGGTTGACATGTGTACTTATTGCAAAGTTATGAGTGTG 4846
NsylMAR1h-c31033    1231:---------------------------------------------------------------------------------------------------- 1231

Nitab4.5_0001338_Tg 4564:GTACTTATTGCAAAGTTATGAGTGTGCGAACAGGTATGCCAAAGCTTGGTCTTTATATGAGCATCCAACTTGAAAGCTTAGGACAGTAGGTCGAGTAATT 4663
Nitab4.5_0001338_Tc 1231:---------------------------------------------------------------------------------------------------- 1231
Nitab4.5_0004525_Sc 1258:---------------------------------------------------------------------------------------------------- 1258
Nitab4.5_0004525_Sg 4847:GGAAAAGGTATGCCAAAGCTTGGTCTTTATATGAGCATCCAACTTGAAAGCTTAGGACAGTAGGTCGAGTAATTCAAGACCTTTGTAACTCCTTTTTAGG 4946
NsylMAR1h-c31033    1231:---------------------------------------------------------------------------------------------------- 1231

Nitab4.5_0001338_Tg 4664:CAAGACCTTTGTACTCCTTTTTGGGACATTGTACAACTCTTATCACTTAACAGTAAACTAGCTGTATGAACTCATAGACGGTTTCTCTTACCCTCAGAAT 4763
Nitab4.5_0001338_Tc 1231:---------------------------------------------------------------------------------------------------- 1231
Nitab4.5_0004525_Sc 1258:---------------------------------------------------------------------------------------------------- 1258
Nitab4.5_0004525_Sg 4947:ACACTGTACAACTCTTATCACTTAAGAATAAAATAGCCGTATGAATTCATAGACGGTTTCTCTTACCCTCAGAGAAGAGGGGTTGCTCTGATGGTAAGCA 5046
NsylMAR1h-c31033    1231:---------------------------------------------------------------------------------------------------- 1231

Nitab4.5_0001338_Tg 4764:GGATGGAATGCTCCTATATTGACCCGTTCCACGAAAATTATCCATTACAAGAGGACCCAAGGGGGTGGCCTAACGGTCAATGTGGGTAAAACCATGGGAG 4863
Nitab4.5_0001338_Tc 1231:---------------------------------------------------------------------------------------------------- 1231
Nitab4.5_0004525_Sc 1258:---------------------------------------------------------------------------------------------------- 1258
Nitab4.5_0004525_Sg 5047:ACCTCCACTTCCAACCAAGAGGTTGTGAGTTCGAGTCACCCCAAGAGCAAGGTGGGGAGTTCTTGGAGGGAAGGATGCCGTTGGAAACAGCTTCTCTACC 5146
NsylMAR1h-c31033    1231:---------------------------------------------------------------------------------------------------- 1231

Nitab4.5_0001338_Tg 4864:AAAAGACTATGTTGCCTAAGAGCCTTGGTGAGTAGAGTTACATGGTGCTTGTGCTGGTAGGAGGTAGCAGGTACTCGTGGGAATAATTGAGGTGTGTGCA 4963
Nitab4.5_0001338_Tc 1231:---------------------------------------------------------------------------------------------------- 1231
Nitab4.5_0004525_Sc 1258:---------------------------------------------------------------------------------------------------- 1258
Nitab4.5_0004525_Sg 5147:CCAGGATAGGGGTAAGGTCTGCGTATACACTACCCTCCCCAGACCCCACTAGTGGGATTATACTGGGTTGTTGTTGTTGTTTCTCTTACCCTCAGAATGG 5246
NsylMAR1h-c31033    1231:---------------------------------------------------------------------------------------------------- 1231

Nitab4.5_0001338_Tg 4964:AGATGGCCCGAACACCACTGTTATAAAAAAATTATCCATTACAAGAAGGAAATAATTAATTGCTTTAATTTTTAAAATCCTTAGTGATTGCAGTGTTGAC 5063
Nitab4.5_0001338_Tc 1231:---------------------------------------------------------------------------------------------------- 1231
Nitab4.5_0004525_Sc 1258:---------------------------------------------------------------------------------------------------- 1258
Nitab4.5_0004525_Sg 5247:ATGGAATGCTCCTATATTGACCAGATTTTCCAAGAAAATTATCCATTACAAGAGGACCCAAAGGTGTGGCCTAACCGTCAATGTGGGTAAAAGTTAAAAA 5346
NsylMAR1h-c31033    1231:---------------------------------------------------------------------------------------------------- 1231

Nitab4.5_0001338_Tg 5064:TTAAAAAATCATATCTAATCCTTTGACCTGAACAGCACCGTGGGAATATTTTGGTGTTGGCAAGCATAGGTGACTTTCTCTGTGTAGTGTCTTGGGCAGT 5163
Nitab4.5_0001338_Tc 1231:---------------------------------------------------------------------------------------------------- 1231
Nitab4.5_0004525_Sc 1258:---------------------------------------------------------------------------------------------------- 1258
Nitab4.5_0004525_Sg 5347:CCATGGAGAAAAGATTATGTTGCCTAAGCCTTGGTGAGTAGAGTTACATGGTGCTTGTGCTGGTAGGAGGTAGCAGGTACTCGTGGGAATAATCGAGGTT 5446
NsylMAR1h-c31033 1231:---------------------------------------------------------------------------------------------------- 1231

Nitab4.5_0001338_Tg 5164:TTCACCCAGTGGACAATGTTTTCGCCTAGGTTATGTTACACGCACACATGCCACTTCTGGGATGTTTCTTTCGGGATATTTTTCATGTGAAATTGTGAGT 5263
Nitab4.5_0001338_Tc 1231:---------------------------------------------------------------------------------------------------- 1231
Nitab4.5_0004525_Sc 1258:---------------------------------------------------------------------------------------------------- 1258
Nitab4.5_0004525_Sg 5447:GTGCAAGATGGCCCGAACACCACTTTTATAAAAAAGAATTATCCATTACAAGAAGGAAATAATTAAATACTTTAATTTTTAAAATCCTTAGTGATTGCAA 5546
NsylMAR1h-c31033    1231:---------------------------------------------------------------------------------------------------- 1231
```

```
Nitab4.5_0001338_Tg 5264:GTGTCCAACCAGTACGGTGGTCTTTTAGTTGTAAATTAGGTGCTAACACCTTTTGAATTCAAGCAGCTTGCTGGCTTTATTAATAGTGTTTTTTGCAACT 5363
Nitab4.5_0001338_Tc 1231:---------------------------------------------------------------------------------------------------- 1231
Nitab4.5_0004525_Sc 1259:------------------------------------------CACCGTGGGAATATTTTGGTGTTGGCAAGCATAG------------------------ 1292
Nitab4.5_0004525_Sg 5547:TGTTGACTTAAAAATTCATGTCTAATCCTTTGACCCGCACAGCACCGTGGGAATATTTTGGTGTTGGCAAGCATAGGTGACTTTCTCTGTGTGGTGTCTT 5646
NsylMAR1h-c31033    1231:---------------------------------------------------------------------------------------------------- 1231

Nitab4.5_0001338_Tg 5364:CTAAGTGGCATATCCTGTAAACATTGGTCTTGTTGTGCACTCCATCTTAAAAGTTTGCGACTAAAGTGGAAATTTGCGTAATCCATGTATGACCTATATG 5463
Nitab4.5_0001338_Tc 1231:---------------------------------------------------------------------------------------------------- 1231
Nitab4.5_0004525_Sc 1292:---------------------------------------------------------------------------------------------------- 1292
Nitab4.5_0004525_Sg 5647:GGGCAGTTTCACCCAGTGGACCATGTTTTCGCCTAGGTTATGTTACACACGCACATGCCACTTCTTGGATGTTTCAGGATATTTTTCATGTGAAATTGTG 5746
NsylMAR1h-c31033    1231:---------------------------------------------------------------------------------------------------- 1231

Nitab4.5_0001338_Tg 5464:CTTTCTGAAGTTTGTAGTTAATTGCTCGGATGAATGAGGATTGCACTTCCTTGCAAAAGCTTTAAAATATATCAGAAACTCATCATTGCAAAAAAAAAAA 5563
Nitab4.5_0001338_Tc 1231:---------------------------------------------------------------------------------------------------- 1231
Nitab4.5_0004525_Sc 1292:---------------------------------------------------------------------------------------------------- 1292
Nitab4.5_0004525_Sg 5747:AGTGTGTCCAACAAGCGCAGTGGCCTTTTAGTTGTAAATTAGGTGCTCACACCTTTTAAATTCAAGCAGCTTGCTGGCTTTATTAATAGTGTTTTTGCAA 5846
NsylMAR1h-c31033    1231:---------------------------------------------------------------------------------------------------- 1231

Nitab4.5_0001338_Tg 5564:AAAAAAAAA------------------------------------------------------------------------------------------- 5572
Nitab4.5_0001338_Tc 1231:---------------------------------------------------------------------------------------------------- 1231
Nitab4.5_0004525_Sc 1292:---------------------------------------------------------------------------------------------------- 1292
Nitab4.5_0004525_Sg 5847:CTCTAAGTGGCATATCCTGTAAACACTGGTCTTGTTATGCACTCCATCTTAGAAGTTTGCAACTAAATTGGAAATTTGCATAATCCATGTATGACCTATA 5946
NsylMAR1h-c31033    1231:---------------------------------------------------------------------------------------------------- 1231

Nitab4.5_0001338_Tg 5572:---------------------------------------------------------------------------------------------------- 5572
Nitab4.5_0001338_Tc 1231:---------------------------------------------------------------------------------------------------- 1231
Nitab4.5_0004525_Sc 1292:---------------------------------------------------------------------------------------------------- 1292
Nitab4.5_0004525_Sg 5947:TGCTTTCTGAAGTTTGTAGGTAATTGCTCAGATGAATGAGAGTTGCACTTCCTGCAAAAGCTTTAAAATATATCAAAAACTCATCATTGCTAAAAAAAAC 6046
NsylMAR1h-c31033    1231:---------------------------------------------------------------------------------------------------- 1231

Nitab4.5_0001338_Tg 5572:---------------------------------------------------------------------------------------------------- 5572
Nitab4.5_0001338_Tc 1231:---------------------------------------------------------------------------------------------------- 1231
Nitab4.5_0004525_Sc 1292:---------------------------------------------------------------------------------------------------- 1292
Nitab4.5_0004525_Sg 6047:TGAAATTGAAAATAGTGACCACTTTCAAAAGATCTTCAAAGAAATAGGAGATCTCTGTGGGGGATGGTTAGCTACAGAAGAAGAAACTGATCTCAAAAAC 6146
NsylMAR1h-c31033    1231:---------------------------------------------------------------------------------------------------- 1231

Nitab4.5_0001338_Tg 5572:---------------------------------------------------------------------------------------------------- 5572
Nitab4.5_0001338_Tc 1231:---------------------------------------------------------------------------------------------------- 1231
Nitab4.5_0004525_Sc 1292:---------------------------------------------------------------------------------------------------- 1292
Nitab4.5_0004525_Sg 6147:CATCTTAAGTGGGCAAGAATCAAAATTGTTGGTGATGGCAGAAAGACTCCCAATGAGGTGGGGATTGAAAGAGACGGAACCAAATTTTTCATCCCAATCT 6246
NsylMAR1h-c31033 1231:---------------------------------------------------------------------------------------------------- 1231

Nitab4.5_0001338_Tg 5572:---------------------------------------------------------------------------------------------------- 5572
Nitab4.5_0001338_Tc 1231:---------------------------------------------------------------------------------------------------- 1231
Nitab4.5_0004525_Sc 1292:---------------------------------------------------------------------------------------------------- 1292
Nitab4.5_0004525_Sg 6247:GGGCTGAAAGAAAGACACGGTATGAGCTTAACACTGGTAAAGGAAAGGCAAGTGGACAATTTGCACAAGGTTCAACTTCGATGAATCAGAGCAGTAAAGT 6346
NsylMAR1h-c31033    1231:---------------------------------------------------------------------------------------------------- 1231

Nitab4.5_0001338_Tg 5572:---------------------------------------------------------------------------------------------------- 5572
Nitab4.5_0001338_Tc 1231:---------------------------------------------------------------------------------------------------- 1231
Nitab4.5_0004525_Sc 1292:---------------------------------------------------------------------------------------------------- 1292
Nitab4.5_0004525_Sg 6347:GTCAAAAGTCAAGCTGAAATCCTATGACCCCGATATGGCTGCACAAGTCATTTTTAATGACCTCTCGTGTGAGATCTCTGATGATACTGGGCGGAAGCCT 6446
NsylMAR1h-c31033    1231:---------------------------------------------------------------------------------------------------- 1231
```

```
Nitab4.5_0001338_Tg 5572:---------------------------------------------------------------------------------------------------- 5572
Nitab4.5_0001338_Tc 1231:---------------------------------------------------------------------------------------------------- 1231
Nitab4.5_0004525_Sc 1292:---------------------------------------------------------------------------------------------------- 1292
Nitab4.5_0004525_Sg 6447:TATATCGAGGAAATGGGAGGGCCTTNNNNNNTCAACTGTTTACCTTGGGATGCCTCTTGGTGCAAGATCCAAATCAAAAGAAATCTGGAATTCAGTCATA 6546
NsylMAR1h-c31033    1231:---------------------------------------------------------------------------------------------------- 1231

Nitab4.5_0001338_Tg 5572:---------------------------------------------------------------------------------------------------- 5572
Nitab4.5_0001338_Tc 1231:---------------------------------------------------------------------------------------------------- 1231
Nitab4.5_0004525_Sc 1292:---------------------------------------------------------------------------------------------------- 1292
Nitab4.5_0004525_Sg 6547:GAAAAGTGTGAGAAGAAGTTGTCAAGATGGAAATCGCAGTACCTATCATTGGGGGGTAGGCTAGTTCTAATCAACTCAGTATTAGACTCTCTACCTACTT 6646
NsylMAR1h-c31033    1231:---------------------------------------------------------------------------------------------------- 1231

Nitab4.5_0001338_Tg 5572:---------------------------------------------------------------------------------------------------- 5572
Nitab4.5_0001338_Tc 1231:---------------------------------------------------------------------------------------------------- 1231
Nitab4.5_0004525_Sc 1292:---------------------------------------------------------------------------------------------------- 1292
Nitab4.5_0004525_Sg 6647:ACATGATGTCTTTGTTCCCAATTCCAGCAGGCGTTCTACAGAGATTGGACAAACTCCGAAGAACTTTCCTTTGGCAAGGCAATAAGGAGAAAAAGGTCTT 6746
NsylMAR1h-c31033    1231:---------------------------------------------------------------------------------------------------- 1231

Nitab4.5_0001338_Tg 5572:---------------------------------------------------------------------------------------------------- 5572
Nitab4.5_0001338_Tc 1231:---------------------------------------------------------------------------------------------------- 1231
Nitab4.5_0004525_Sc 1292:---------------------------------------------------------------------------------------------------- 1292
Nitab4.5_0004525_Sg 6747:CGATTTAGTCAATTGGAAGACGCTAACAATGGATAAAAAACAAGGTGGAATGGGGATTAGGAATTTGAAGAACCAAAGCAAGGCTCTTAGAATTAAATGG 6846
NsylMAR1h-c31033    1231:---------------------------------------------------------------------------------------------------- 1231

Nitab4.5_0001338_Tg 5572:---------------------------------------------------------------------------------------------------- 5572
Nitab4.5_0001338_Tc 1231:---------------------------------------------------------------------------------------------------- 1231
Nitab4.5_0004525_Sc 1292:---------------------------------------------------------------------------------------------------- 1292
Nitab4.5_0004525_Sg 6847:TTATGGAAGTACTCTAAAGAACCCCAATCTTTATGGTCCAAAGTGATCAAAGCAAAGTATGGTGAAGAAAACAACTGGGTGTCAAAAAGAGTCAGAACAT 6946
NsylMAR1h-c31033 1231:---------------------------------------------------------------------------------------------------- 1231

Nitab4.5_0001338_Tg 5572:---------------------------------------------------------------------------------------------------- 5572
Nitab4.5_0001338_Tc 1231:---------------------------------------------------------------------------------------------------- 1231
Nitab4.5_0004525_Sc 1292:---------------------------------------------------------------------------------------------------- 1292
Nitab4.5_0004525_Sg 6947:CACATGGAGTAAGTGTGTGGAAATCCATCAGAGAACCTTGGCCAATAATGAAGAATCATTCCTCCATAAGAGTGAACAACGGAAGGAACACATCATTTTG 7046
NsylMAR1h-c31033    1231:---------------------------------------------------------------------------------------------------- 1231

Nitab4.5_0001338_Tg 5572:---------------------------------------------------------------------------------------------------- 5572
Nitab4.5_0001338_Tc 1231:---------------------------------------------------------------------------------------------------- 1231
Nitab4.5_0004525_Sc 1292:---------------------------------------------------------------------------------------------------- 1292
Nitab4.5_0004525_Sg 7047:GAGTGATAACTGGTTAGGAATTGGAAGCTTAAAGGAAAGATACCCAGATATGTTCGTTGTTGCACAAAACCAGCATAAGACAGTAGCTGAAATGAGGAGT 7146
NsylMAR1h-c31033    1231:---------------------------------------------------------------------------------------------------- 1231

Nitab4.5_0001338_Tg 5572:---------------------------------------------------------------------------------------------------- 5572
Nitab4.5_0001338_Tc 1231:---------------------------------------------------------------------------------------------------- 1231
Nitab4.5_0004525_Sc 1292:---------------------------------------------------------------------------------------------------- 1292
Nitab4.5_0004525_Sg 7147:TGTCATGGATGGGAAATAGCTCTAAGAAGAGAGCTGAATGACTGGGAGATAATAAGACTAACTGACCTCTACAAAGAGCTGGAAGCATTTACAGGATTAC 7246
NsylMAR1h-c31033    1231:---------------------------------------------------------------------------------------------------- 1231

Nitab4.5_0001338_Tg 5572:---------------------------------------------------------------------------------------------------- 5572
Nitab4.5_0001338_Tc 1231:---------------------------------------------------------------------------------------------------- 1231
Nitab4.5_0004525_Sc 1292:---------------------------------------------------------------------------------------------------- 1292
Nitab4.5_0004525_Sg 7247:AGGAAGGTTTGGATTCAATATGGTGGAAGAGGCACAACAGAGGGGTTTACCGAGTGAAGGATGCATATAAGATTTTGAATCATGATAATCAACAGGTAGA 7346
NsylMAR1h-c31033    1231:---------------------------------------------------------------------------------------------------- 1231
```

```
Nitab4.5_0001338_Tg 5572:---------------------------------------------------------------------------------------------------- 5572
Nitab4.5_0001338_Tc 1231:---------------------------------------------------------------------------------------------------- 1231
Nitab4.5_0004525_Sc 1292:---------------------------------------------------------------------------------------------------- 1292
Nitab4.5_0004525_Sg 7347:CACATGGCCTTGGAAACATATATGGAAAACAAAGATTCCATACAAGGTAGCTTGCTTCACTTGGCTACTAGCAAAGGAGGGGTTTTAACCCAGGATAATC 7446
NsylMAR1h-c31033    1231:---------------------------------------------------------------------------------------------------- 1231

Nitab4.5_0001338_Tg 5572:---------------------------------------------------------------------------------------------------- 5572
Nitab4.5_0001338_Tc 1231:---------------------------------------------------------------------------------------------------- 1231
Nitab4.5_0004525_Sc 1292:---------------------------------------------------------------------------------------------------- 1292
Nitab4.5_0004525_Sg 7447:TCATAAAAAGGGGAATATCTCTGTGTTCAAGATGTTTTCTGTGTGGAGAAAATGCAGAAACTGTCAACCATTTGTTTCTACATTGCAAGATTACAGACCA 7546
NsylMAR1h-c31033    1231:---------------------------------------------------------------------------------------------------- 1231

Nitab4.5_0001338_Tg 5572:---------------------------------------------------------------------------------------------------- 5572
Nitab4.5_0001338_Tc 1231:---------------------------------------------------------------------------------------------------- 1231
Nitab4.5_0004525_Sc 1292:---------------------------------------------------------------------------------------------------- 1292
Nitab4.5_0004525_Sg 7547:CTATGGAAAATCTTTATAAGTCTTAGGGGTATTTCATGGTCAATGTCATACAAGATTAAAGATGTCATTTATAGTTGGGAAGTAGCTGGAGCTGAAGCAA 7646
NsylMAR1h-c31033    1231:---------------------------------------------------------------------------------------------------- 1231

Nitab4.5_0001338_Tg 5572:---------------------------------------------------------------------------------------------------- 5572
Nitab4.5_0001338_Tc 1231:---------------------------------------------------------------------------------------------------- 1231
Nitab4.5_0004525_Sc 1292:---------------------------------------------------------------------------------------------------- 1292
Nitab4.5_0004525_Sg 7647:CTAGCAGAGATAGATGGAGGACTGTTCCGGCTTGTATTTGGTGGACAGTCTGGAGGGAAAGGAACACTAGATGTTTTGAAGACAGAAGCAATCCACTGCA 7746
NsylMAR1h-c31033    1231:---------------------------------------------------------------------------------------------------- 1231

Nitab4.5_0001338_Tg 5572:---------------------------------------------------------------------------------------------------- 5572
Nitab4.5_0001338_Tc 1231:---------------------------------------------------------------------------------------------------- 1231
Nitab4.5_0004525_Sc 1292:---------------------------------------------------------------------------------------------------- 1292
Nitab4.5_0004525_Sg 7747:GAAGATCAAACTCAATTGTATTCTTCTATTTTGTTTTTGGTGCAAACAGATGTACACAGAAGATACATTGACAATCATAGACATACTAGGATCCTGCTAG 7846
NsylMAR1h-c31033    1231:---------------------------------------------------------------------------------------------------- 1231

Nitab4.5_0001338_Tg 5573:------------------------------------------------------------------------------------------------TGAA 5576
Nitab4.5_0001338_Tc 1231:---------------------------------------------------------------------------------------------------- 1231
Nitab4.5_0004525_Sc 1292:---------------------------------------------------------------------------------------------------- 1292
Nitab4.5_0004525_Sg 7847:GTTTTAAATTAGAATATCTTCAAATTCTCTCTATGTAAATTTGGTTTTCAGTGCAACCTATGTATGATTTTTATAATATATACAATAGTTACCAATTCAA 7946
NsylMAR1h-c31033    1231:---------------------------------------------------------------------------------------------------- 1231

                                                     T4CAPS&AS-F
Nitab4.5_0001338_Tg 5577:ATTGAAAATAGTGACCAATTTAATCTCAAGGGAACATCACTGTAATGATTTCAGGTTTAAATCCATCTATTATCGGGTGCTTCAGTGGAATATGTGCTTT 5676
Nitab4.5_0001338_Tc 1232:------------------------------------------------------GTTTAAATCCATCTATTATCGGGTGCTTCAGTGGAATATGTGCTTT 1277
Nitab4.5_0004525_Sc 1293:-------------------------------------------------------------------------------------TGGAATATGTGCTTT 1307
Nitab4.5_0004525_Sg 7947:AAAAAAAATAGTGACCACTTTAATCTCAAGGGAACATCACTGTAATGATTTCAGGTTTAAATCCATCTGTTATTGGGGGCTTCAGTGGAATATGTGCTTT 8046
NsylMAR1h-c31033    1232:------------------------------------------------------GTTTAAATCCATCTATTATTGGGGGCTTCAGTGGAATATGTGCTTT 1277
                                                                                   ................... ... .......***************

Nitab4.5_0001338_Tg 5677:TATGGGCGTCGCGGCAACATTTGTCTCCGCAAAGATGGTCAAGCACCTTGGCATCTTAAGGGTTGGTTCTGCTTCTTATATGTTTCTTGTTCTTGTCAGA 5776
Nitab4.5_0001338_Tc 1278:TATGGGCGTCGCGGCAACATTTGTCTCCGCAAAGATGGTCAAGCACCTTGGCATCTTAAGG--------------------------------------- 1338
Nitab4.5_0004525_Sc 1308:AATGGGCGTCGCAGCAACATTTGTCTCTGCAAAGATGGTCAAGCACCTTGGCATCTTAAGG--------------------------------------- 1368
Nitab4.5_0004525_Sg 8047:AATGGGCGTCGCAGCAACATTTGTCTCTGCAAAGATGGTCAAGCACCTTGGCATCTTAAGGGTTGGTTCTGCTTCTTATATGTTTCTTGTTCTTGTCAGA 8146
NsylMAR1h-c31033    1278:AATGGGCGTCGCAGCAACATTTGTCTCTGCAAAGATGGTCAAGCACCTTGGCATCTTAAGG--------------------------------------- 1338
                         .***********.**************.*********************************

Nitab4.5_0001338_Tg 5777:AATAGTGTCCTCTTATCATTCACAGTCTGTGTCTAACTTTCATGAATTTTAAAATATCAGGCTGGAGCTGCTGGCCTAATATTCCAGGCTTCGCTTCTGA 5876
Nitab4.5_0001338_Tc 1339:------------------------------------------------------------GCTGGAGCTGCTGGCCTAATATTCCAGGCTTCGCTTCTGA 1378
Nitab4.5_0004525_Sc 1369:------------------------------------------------------------GCTGGAGCTGCTGGCCTAATTTTCCAGGCTTCGCTTCTGA 1408
Nitab4.5_0004525_Sg 8147:AATAGTGTCCTCTTATCATTCACAGTCTGGGTCTAACTTTCATGAATTTTAAAATATCAGGCTGGAGCTGCTGGCCTAATTTTCCAGGCTTCGCTTCTGA 8246
NsylMAR1h-c31033    1339:------------------------------------------------------------GCTGGAGCTGCTGGCCTAATTTTCCAGGCTTCGCTTCTGA 1378
                                                                                     ********************.*******************
```

```
                                                           T4CAPS&AS-R (compl)
Nitab4.5_0001338_Tg 5877:CCACAGCTGTCGCTGTCTACTGGAGTGGATCTCTTTCTCAGCAGACTCCCGTTTTCTTTTTCTTGGCTTTAGTTGTAAGTTACTGCTCTTGTTATTTTTG 5976
Nitab4.5_0001338_Tc 1379:CCACAGCTGTCGCTGTCTACTGGAGTGGATCTCTTTCTCAGCAGACTCCCGTTTTCTTTTTCTTGGCTTTAGTT-------------------------- 1452
Nitab4.5_0004525_Sc 1409:CCACAGCTGTTGCTGTCTACTGGAGTGGATCTCTTTCTCAGCAGACTCCCGTTTTCTTTTTCTTGGCTTTAGTT-------------------------- 1482
Nitab4.5_0004525_Sg 8247:CCACAGCTGTTGCTGTCTACTGGAGTGGATCTCTTTCTCAGCAGACTCCCGTTTTCTTTTTCTTGGCTTTAGTTGTAAGTTATTGCTCTTGTTATTTTTG 8346
NsylMAR1h-c31033    1379:CCACAGCTGTTGCTGTCTACTGGAGTGGATCTCTTTCTCAGCAGACTCCCGTTTTCTTTTTCTTGGCTTTAGTT-------------------------- 1452
                         **********.***************************************************************

Nitab4.5_0001338_Tg 5977:CTTTAAATGAGAATTTTTTGTGGCTAACTGGAGTAGCCTTTTATTTGCATTTAAAGAGGCTATTTTTGAAAGTGCTTTATCTTTCGACGGATTGCTTCAT 6076
Nitab4.5_0001338_Tc 1452:---------------------------------------------------------------------------------------------------- 1452
Nitab4.5_0004525_Sc 1482:---------------------------------------------------------------------------------------------------- 1482
Nitab4.5_0004525_Sg 8347:CTTTAAATGAGAATTTTTTGTGGCCAACTATATAACCTTTTATTTGCATTTAAAGAACCTATTTTTGAAAGTGCTTTATCTTTCGACAGATTGCTTCATG 8446
NsylMAR1h-c31033    1452:---------------------------------------------------------------------------------------------------- 1452

Nitab4.5_0001338_Tg 6077:GAGAATTTTATATTCATCTAGTTTCGTTTGCATGCTCTGGTACTTATATGATTTAGTC-TGTAAAGGTGATCTGAAATTGGATAATTGTTATCTTAAGTT 6175
Nitab4.5_0001338_Tc 1452:---------------------------------------------------------------------------------------------------- 1452
Nitab4.5_0004525_Sc 1482:---------------------------------------------------------------------------------------------------- 1482
Nitab4.5_0004525_Sg 8447:AGAATTTTATATTCATCTAGTTTCGTTGGCATGCTCTGGTACTTGTATGATTTAGTGACTGTAAAGATGATCTGAAATTGGATAATTGTTATCGTAAGTT 8546
NsylMAR1h-c31033    1452:---------------------------------------------------------------------------------------------------- 1452


Nitab4.5_0001338_Tg 6176:CAAGAAGACTTATGTTGACTATTTCACAGGTATTGTCAAGACTGGGACACATGTCCTATGATGTCATTGGGCAACAGATTCTACAAACTGGAATACCTGC 6275
Nitab4.5_0001338_Tc 1453:-----------------------------GTATTGTCAAGACTGGGACACATGTCCTATGATGTCATTGGGCAACAGATTCTACAAACTGGAATACCTGC 1523
Nitab4.5_0004525_Sc 1483:-----------------------------GTATTGTCAAGACTGGGACACATGTCCTATGATGTCATTGGGCAACAGATTCTACAAACTGGAATACCTGC 1553
Nitab4.5_0004525_Sg 8547:CAAGAAGACTTATGTTGACTATTTCACAGGTATTGTCAAGACTGGGACACATGTCCTATGATGTCATTGGGCAACAGATTCTACAAACTGGAATACCTGC 8646
NsylMAR1h-c31033    1453:-----------------------------GTATTGTCAAGACTGGGACACATGTCCTATGATGTCATTGGGCAACAGATTCTACAAACTGGAATACCTGC 1523
                                                      ***********************************************************************

Nitab4.5_0001338_Tg 6276:ATCTAAGGCTAATCTTATCGGAACAACCGAGGTTGCTGTTGCAAGTTTAGCAGAATCAATCATGTTAGGAGTTGCAATAATTGTTAATGATGTCTCACAT 6375
Nitab4.5_0001338_Tc 1524:ATCTAAGGCTAATCTTATCGGAACAACCGAGGTTGCTGTTGCAAGTTTAGCAGAATCAATCATGTTAGGAGTTGCAATAATTGTTAATGATGTCTCACAT 1623
Nitab4.5_0004525_Sc 1554:ATCTAAGGCTAATCTTGTAGGAACAACCGAGGTTGCTGTTGCAAGTTTAGCAGAATCAATCATGTTAGGAGTTGCAATAATTGTTAATGATGTCTCACAT 1653
Nitab4.5_0004525_Sg 8647:ATCTAAGGCTAATCTTGTAGGAACAACCGAGGTTGCTGTTGCAAGTTTAGCAGAATCAATCATGTTAGGAGTTGCAATAATTGTTAATGATGTCTCACAT 8746
NsylMAR1h-c31033    1524:ATCTAAGGCTAATCTTGTAGGAACAACCGAGGTTGCTGTTGCAAGTTTAGCAGAATCAATCATGTTAGGAGTTGCAATAATTGTTAATGATGTCTCACAT 1623
                         ****************.*.*********************************************************************************

Nitab4.5_0001338_Tg 6376:TTTGGATTTCTAGCGACACTTTCCCTTGTATCAGTAGTTGGGGCAGCATGTCTATACTGTAGATGGTTGGAAAATCCGACAGATACACAAAGGACTCTTT 6475
Nitab4.5_0001338_Tc 1624:TTTGGATTTCTAGCGACACTTTCCCTTGTATCAGTAGTTGGGGCAGCATGTCTATACTGTAGATGGTTGGAAAATCCGACAGATACACAAAGGACTCTTT 1723
Nitab4.5_0004525_Sc 1654:TTTGGATTTCTAGCGACACTTTCCCTTGTATCAGTAGTTGGGGCAGCATGTCTATACTGTAGATGGTTGGAAAGTCCGACAGATACACAAAGGACTCTTT 1753
Nitab4.5_0004525_Sg 8747:TTTGGATTTCTAGCGACACTTTCCCTTGTATCAGTAGTTGGGGCAGCATGTCTATACTGTAGATGGTTGGAAAGTCCGACAGATACACAAAGGACTCTTT 8846
NsylMAR1h-c31033    1624:TTTGGATTTCTAGCGACACTTTCCCTTGTATCAGTAGTTGGGGCAGCATGTCTATACTGTAGATGGTTGGAAAGTCCGACAGATACACAAAGGACTCTTT 1723
                         *************************************************************************.**************************

Nitab4.5_0001338_Tg 6476:TCTCTTTTCGCCCTCATTTTTGA                                                                              6498
Nitab4.5_0001338_Tc 1724:TCTCTTTTCGCCCTCATTTTTGA                                                                              1746
Nitab4.5_0004525_Sc 1754:TCTCTTTTAGCCCTCATTTTTGA                                                                              1776
Nitab4.5_0004525_Sg 8847:TCTCTTTTAGCCCTCATTTTTGA                                                                              8869
NsylMAR1h-c31033    1724:TCTCTTTTAGCCCTCATTTTTGA                                                                              1746
                         ********.**************
```

Supplementary Figure S2. Nucleotide sequence alignment of *NtMAR1T* genomic and cDNA sequences (Nitab4.5_0001338_Tg and Nitab4.5_0001338_Tc, respectively), *NtMAR1S* genomic and cDNA sequences (Nitab4.5_0004525_Sg and Nitab4.5_0004525_Sc, respectively), and *Nicotiana sylvestris MAR1* homolog cDNA sequence (NsylMAR1h-c31033). Asterisks show conserved sites throughout the five sequences, and dots show partially conserved sites. CRISPR/Cas9 target sequences were selected in exons 5, 9, and 11/12 (exon 11 in Nitab4.5_0001338_Tg and exon 12 in Nitab4.5_0004525_Sg), which are highlighted in yellow. Target sequences are highlighted by green (target sequence) and magenta (PAM), including target 1 that we designed but omitted from the experiments. Primers used for CAPS and amplicon-seq analyses (Supplementary Table S1) are highlighted in blue.

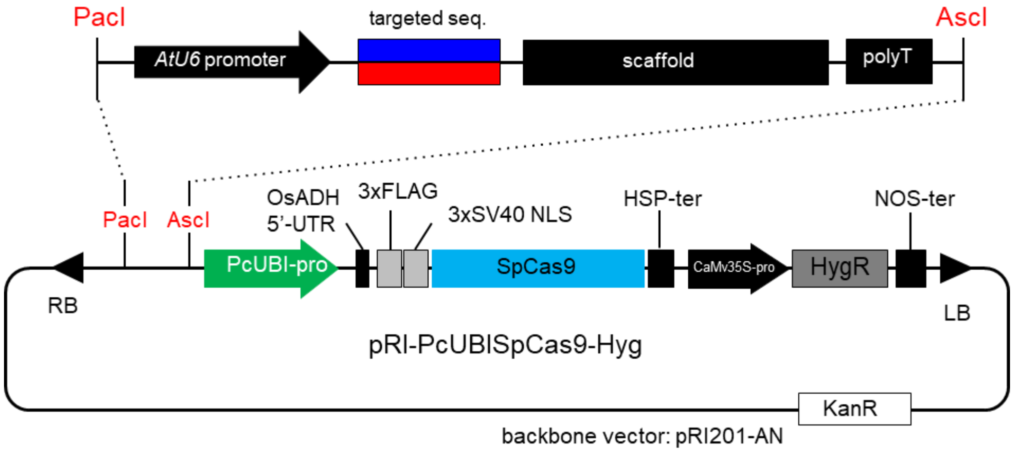

Supplementary Figure S3. CRISPR-*SpCas9* expression construct. SpCas9, plant-optimized Cas9 coding region from *Streptococcus pyogenes*; 3xFLAG, tandemly arranged three FLAG tags; 3xSV40 NLS, tandemly arranged three SV40 nuclear logalization signal; OsADH 5′-UTR, *Oryza sativa* Alcohol Dehydrogenase gene 5′ untranslated region; HSP-ter, *Arabidopsis thaliana* Heat Shock Protein 18.2 gene terminator; HygR, hygromycin resistance gene; CaMV35S pro, cauliflower mosaic virus 35S promoter; NOS-ter, nopaline synthase gene terminator; KanR, Kanamycin resistance gene; AtU6 promoter, *Arabidopsis thaliana* U6 RNA promoter region; scaffold, sgRNA scaffold region; polyT, class 3 terminator.

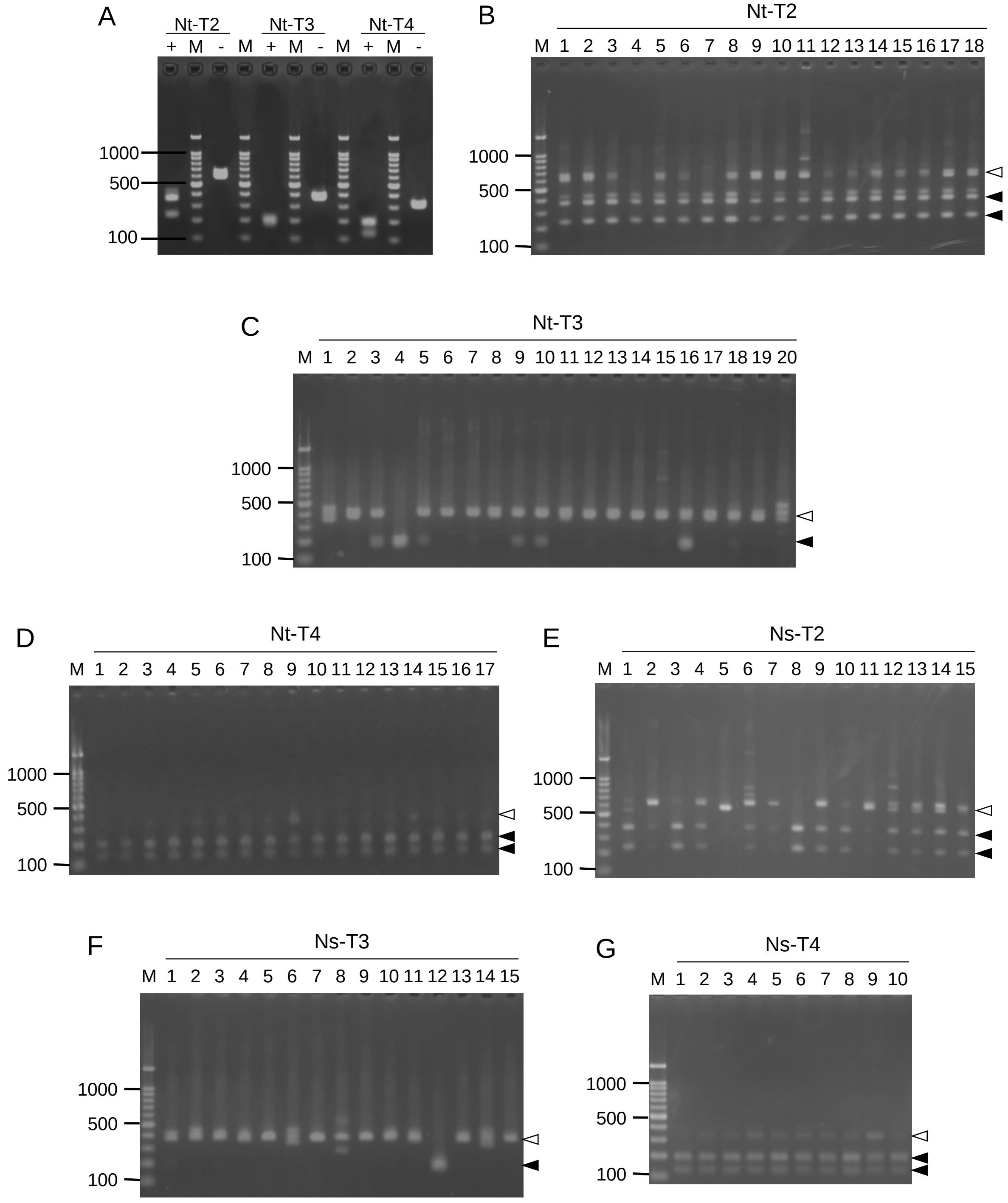

Supplementary Figure S4. Detection of CRISPR/*Cas9*-mediated mutagenesis by the CAPS analysis of *MAR1/RTS3* homologs. (A) Analysis of wild type *N. tabacum* DNA in all three targets. (-) and (+) denote the PCR products before and after restriction enzyme treatment, respectively. Individual transgenic lines of *N. tabacum* (B-D) and *N. sylvestris* (E-G) for target 2 (B and E), target 3 (B and F), and target 4 (C and G) were analyzed. Only the analyses of PCR products treated with restriction enzymes are shown. Open and closed arrowheads indicate uncleaved and cleaved fragments, respectively. M, Molecular marker. Lane numbers correspond to plant line numbers.

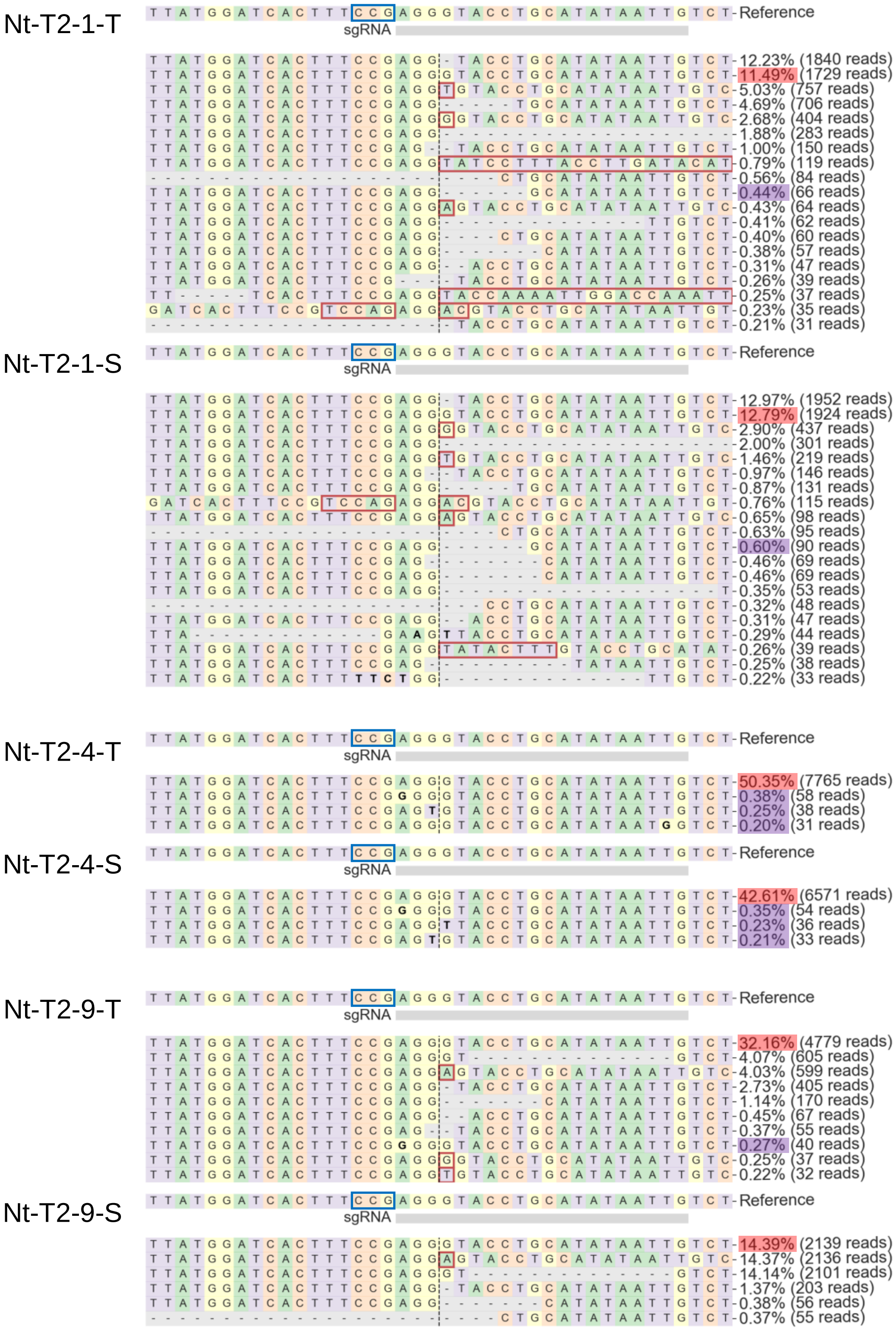

Supplementary Figure S5 (continues)

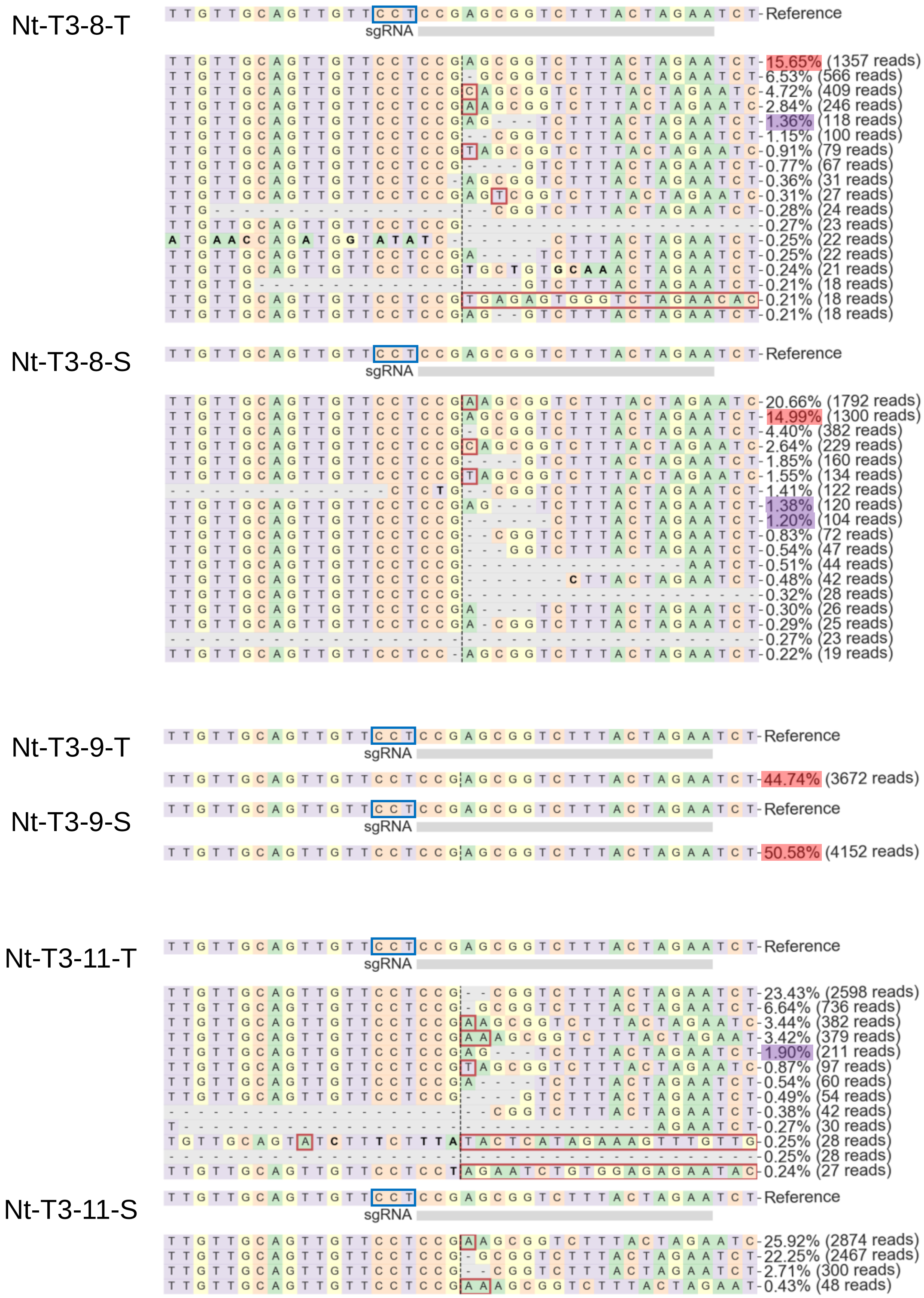

Supplementary Figure S5 (continues)

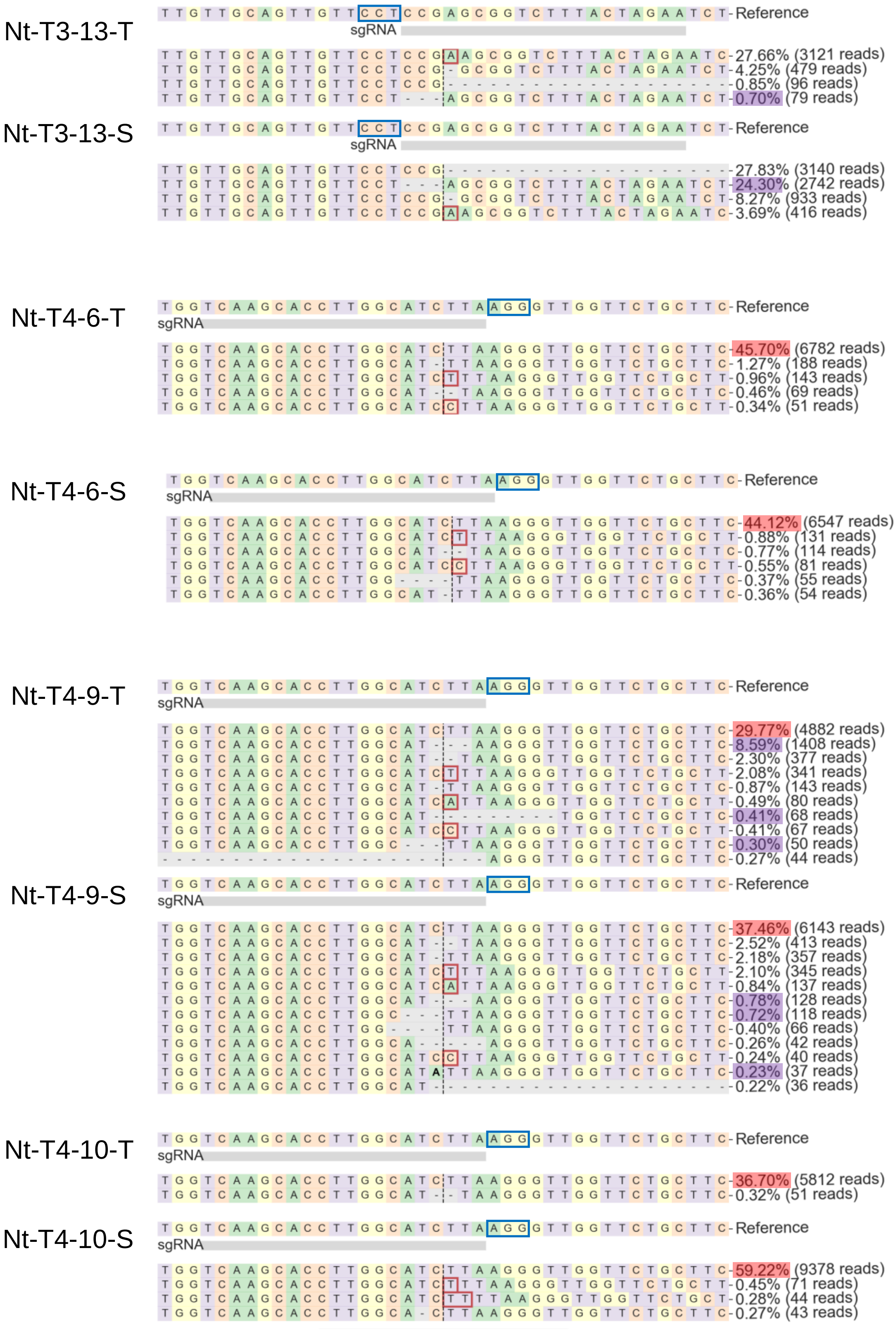

Supplementary Figure S5 (continues)

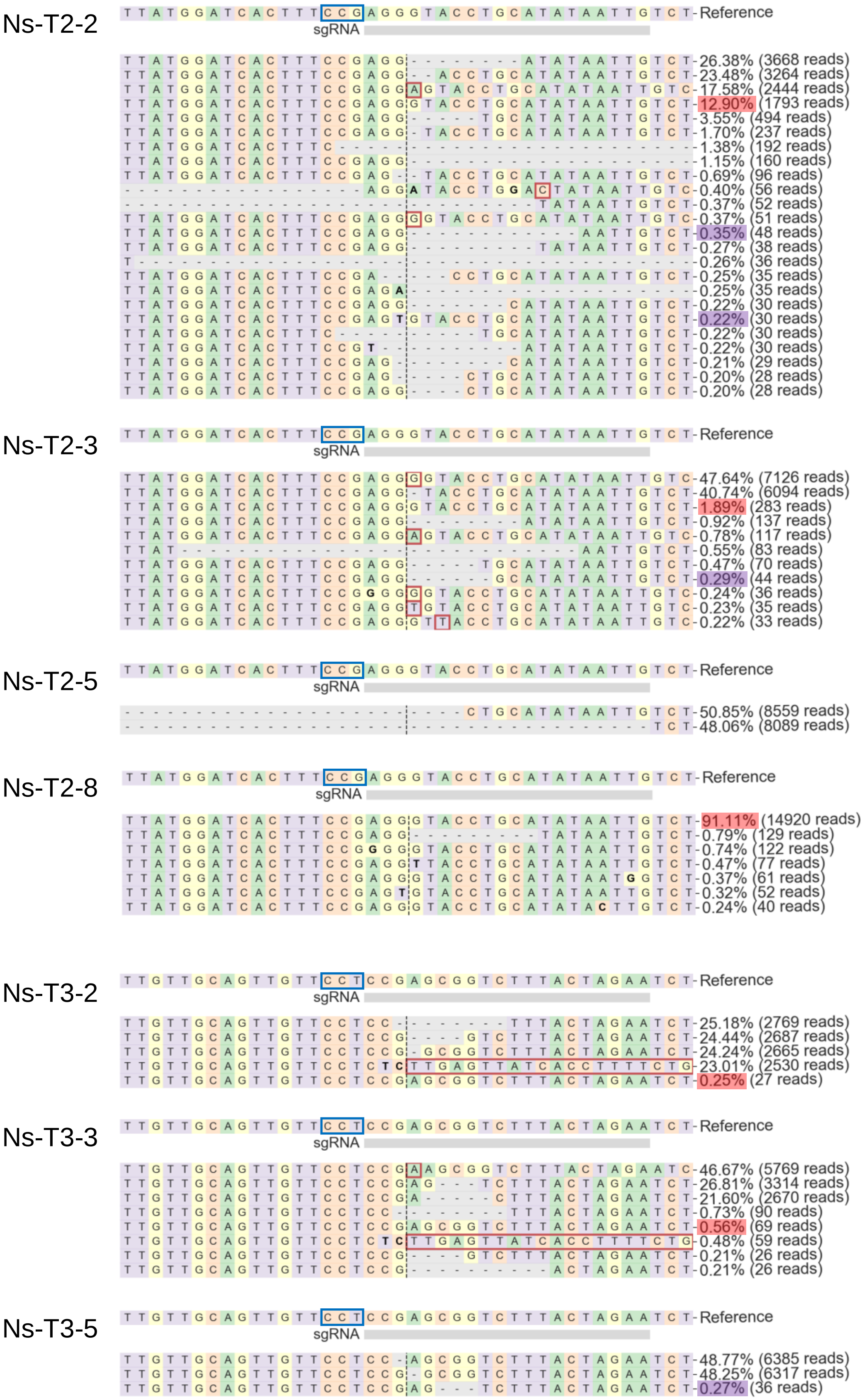

Supplementary Figure S5 (continues)

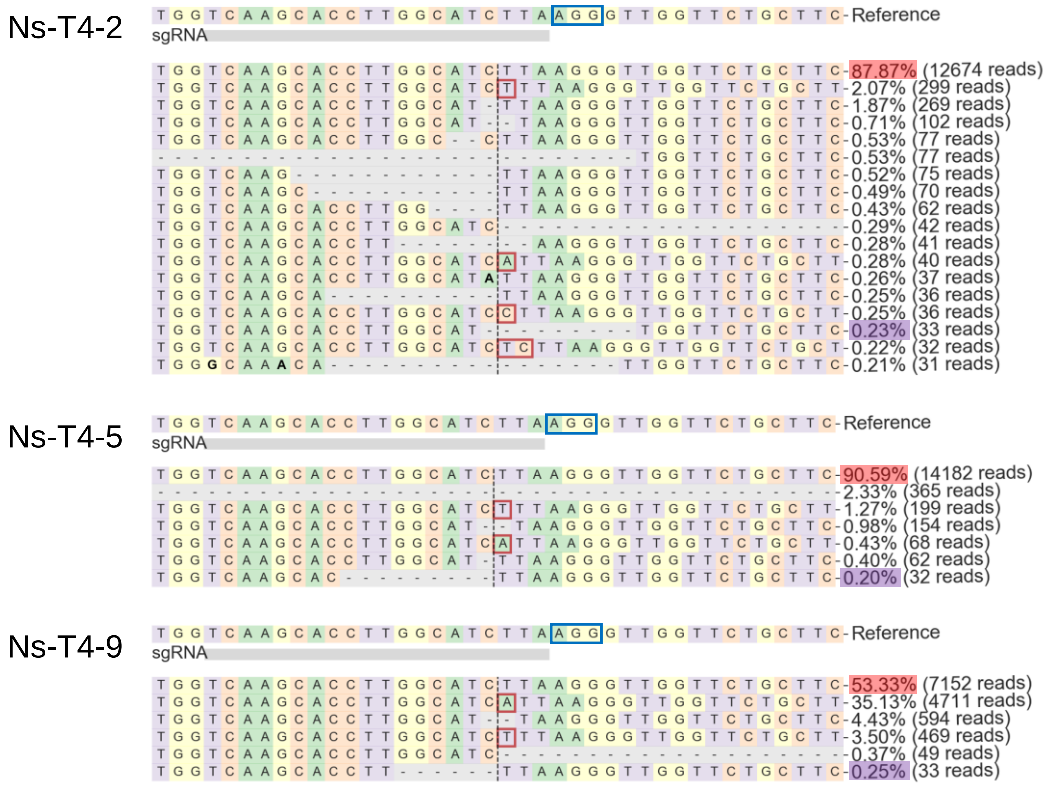

Supplementary Figure S5. Alignment of representative amplicon sequencing reads. Amplicon sequencing reads were aligned with the reference sequence for each of the selected transgenic lines. The T- and S-genome copies are separately shown for *N. tabacum*. The shaded thick lines below the Reference sequence indicate the target region; blue boxes indicate PAM site, bold letters indicate base substitutions, red boxes indicate base insertions, and dashes (-) indicate deletions. Vertical dashed lines indicate predicted cleavage position. Red and purple highlights of percentages denote those of wild-type sequences and non-frame shifting mutations [base substitusions and small (3–9 bps) deletions].

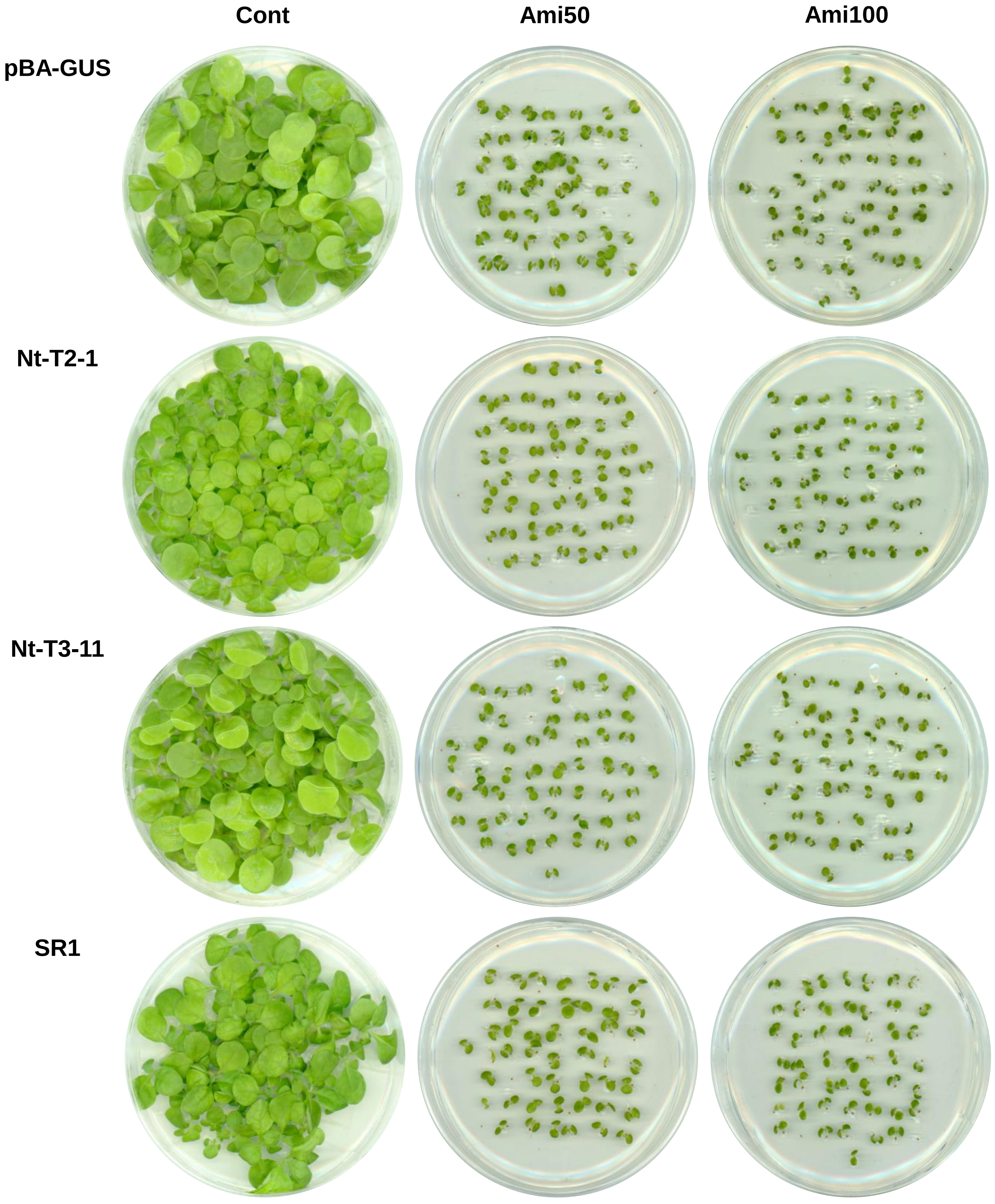

Supplementary Figure S6 (continues)

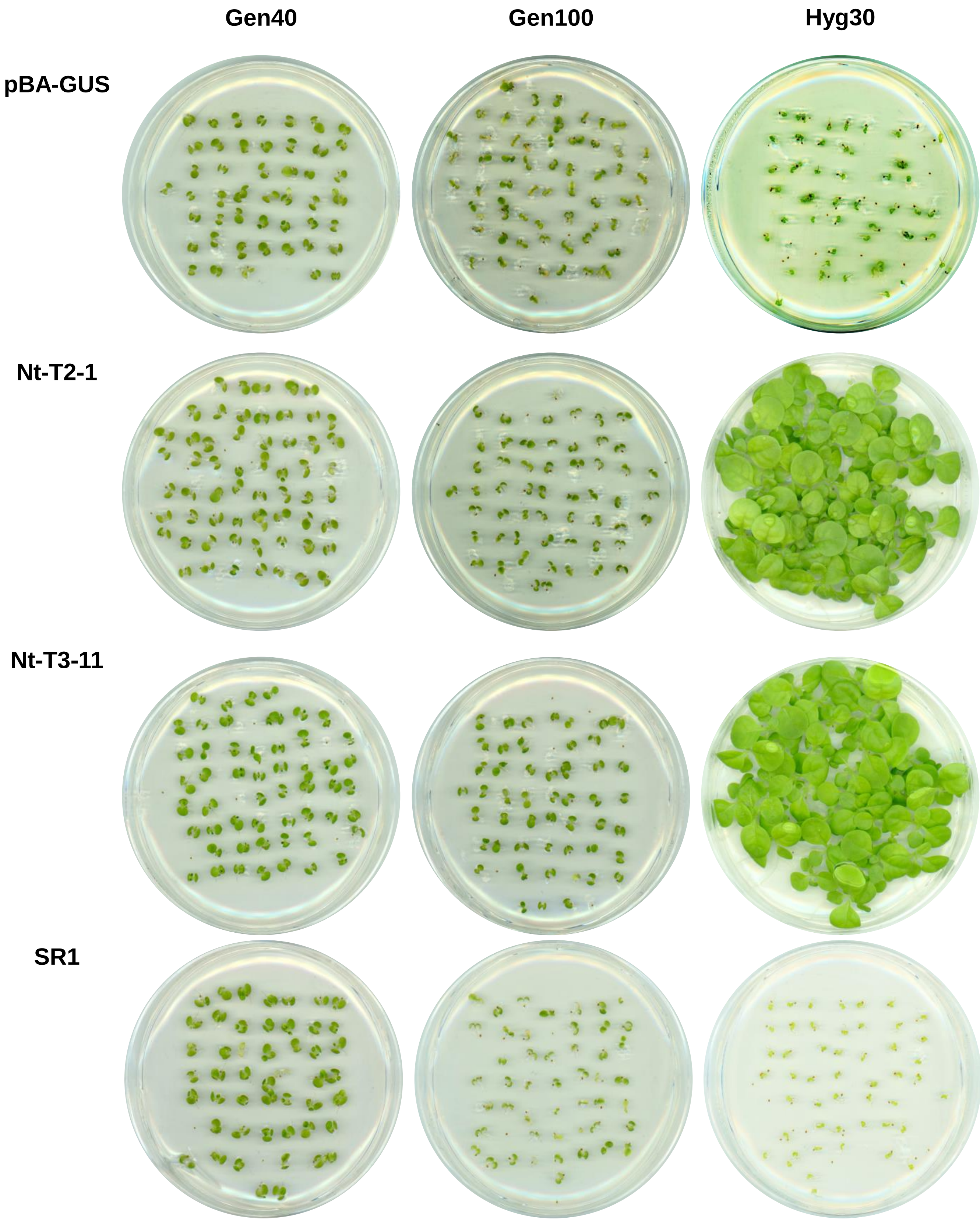

Supplementary Figure S6 (continues)

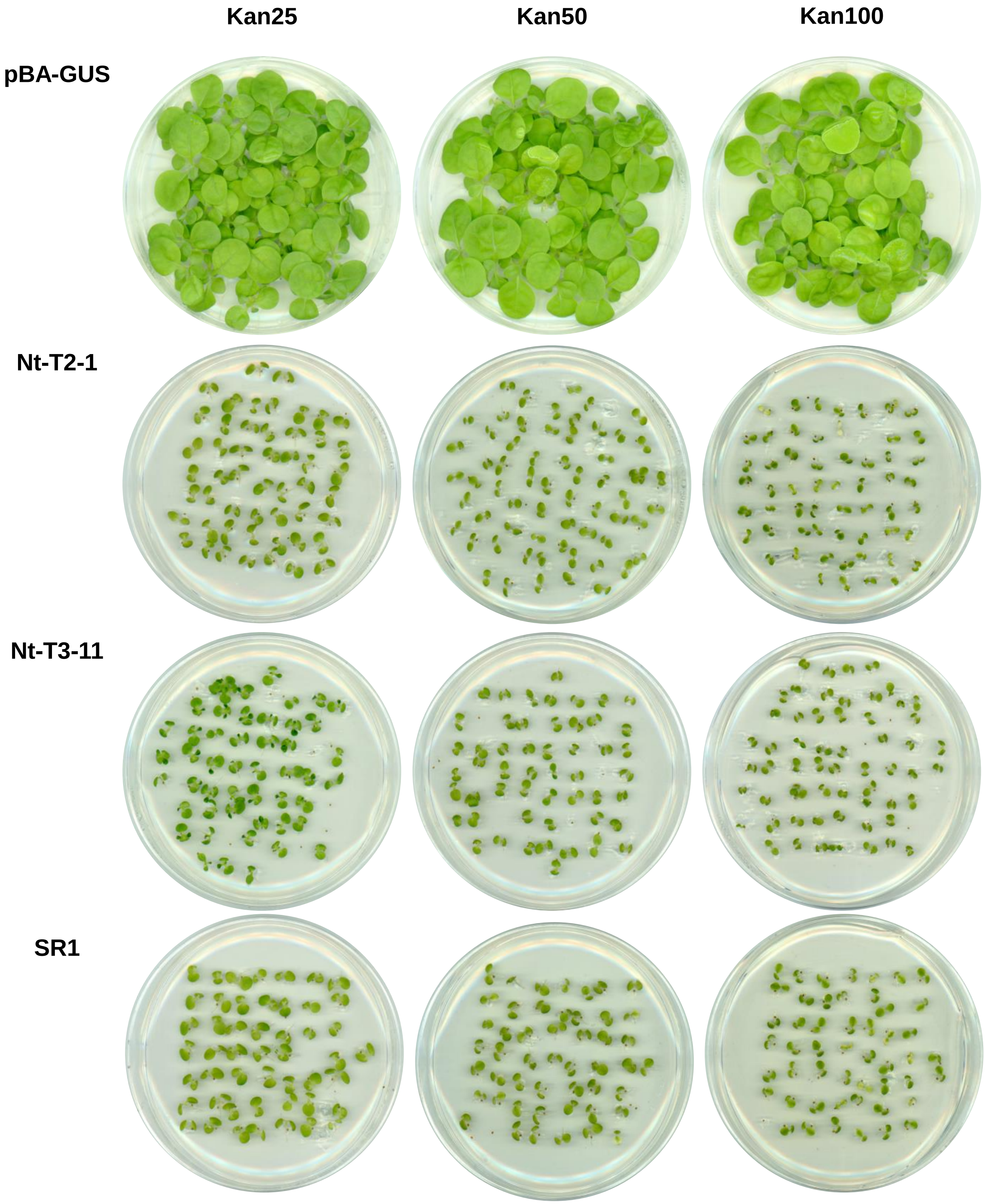

Supplementary Figure S6 (continues)

Supplementary Figure S6. Sensitivity to different antibiotics of T_1 generation seedlings from Nt-T2-1 and Nt-T3-11 lines. pBA-GUS, transgenic tobacco with a kanamycin resistance gene; SR1, non-transformed control; Cont, control media containing no antibiotics; Ami50 and Ami100, media containing 50 and 100 mg/L amikacin, respectively; Gen40 and Gen100, media containing 40 and 100 mg/L gentamycin, respectively; Hyg30, media containing 30 mg/L hygromycin; Kan25, Kan50, and Kan100, media containing 25, 50, and 100 mg/L Kanamycin, respectively.
